# Supplementary material for: Systematic Review on Influenza Burden in Emerging Markets in 2018–2023—An Evidence Update to Guide Influenza Vaccination Recommendations
Source: Vaccines (Basel). 2024 Nov 2;12(11):1251. doi: 10.3390/vaccines12111251 (PMC11599016; doi:10.3390/vaccines12111251)
Supplement: Supplementary file 1 [file vaccines-12-01251-s001.zip › vaccines-3257350-supplementary.pdf]

Supplementary material manuscript ‘Systematic Review on Influenza Burden in Emerging Markets 2018-2023 – An Evidence Update to Guide Influenza Vaccination Recommendations’

Table S1. Search strategy clinical burden of influenza and influenza-like illness infection.

**OVID EMBASE (database search conducted on 8 September 2023)**			
**#**	**Concept group**	**Search terms**	**Hits**
1	Influenza	exp Influenza/ or exp "Influenza A Virus"/ or exp "Influenza B Virus"/	
2		(Influenza or flu or "influenza like illness" or ILI or influenza-associated or influenza-related or influenza associated or influenza related or ILI-associated or ILI-related or ILI associated or ILI related or influenza like illness associated or influenza like illness related).tw,kf	
3	Outcomes	exp hospitalization/ or exp mortality/ or exp outpatient/ or infection/	
4		(case* or infection* or emergency room visit* or ER visit* or hospital* or hospitalization or hospitalisation or mortality or death or ICU or intensive care or critical care or outpatient or mechanical ventilation or supplemental oxygen).tw,kf	
5	Effect measurements	exp incidence/ or exp prevalence/ or exp "disease burden"/	
6		(incidence or incidence rate or prevalence or burden).tw,kf	
7	Regions of interest	asia/ or middle east/ or Africa/ or Latin America/ NOT China/ NOT Japan/	
8		(Asia or middle east or Africa or Latin America).tw,kf	
9		("Afghanistan" or "Armenia" or "Azerbaijan" or "Bahrain" or "Bangladesh" or "Bhutan" or "Brunei" or "Cambodia" or "Cyprus" or "Georgia" or "India" or "Indonesia" or "Iran" or "Iraq" or "Israel" or "Jordan" or "Kazakhstan" or "Kuwait" or "Kyrgyzstan" or "Laos" or "Lebanon" or "Malaysia" or "Maldives" or "Mongolia" or "Myanmar" or "Nepal" or "north korea" or "Oman" or "Pakistan" or "Palestine" or "Philippines" or "Qatar" or "Russia" or "saudi arabia" or "Singapore" or "south korea" or "sri lanka" or "Syria" or "Taiwan" or "Tajikistan" or "Thailand" or "Timor-Leste" or "Turkey" or "Turkmenistan" or "united arab emirates" or "UAE" or "Uzbekistan" or "Vietnam" or "Yemen" or "Algeria" or "Angola" or "Benin" or "Botswana" or "burkina faso" or "Burundi" or "Cabo" or "Verde" or "Cameroon" or "central african republic" or "Chad" or "Comoros" or "Congo" or "Democratic Republic of the Congo" or "cote d ivoire" or "Djibouti" or "Egypt" or "equatorial guinea" or "Eritrea" or "Eswatini" or "Ethiopia" or "Gabon" or "Gambia" or "Ghana" or "Guinea" or "Guinea-Bissau" or "Kenya" or "Lesotho" or "Liberia" or "Libya" or "Madagascar" or "Malawi" or "Mali" or "Mauritania" or "Mauritius" or "Morocco" or "Mozambique" or "Namibia" or "Niger" or "Nigeria" or "Rwanda" or "Sao Tome and Principe" or "Senegal" or "Seychelles" or "sierra leone" or "Somalia" or "south africa" or "south sudan" or "Sudan" or "Tanzania" or "Togo" or "Tunisia" or "Uganda" or "Zambia" or "Zimbabwe" or "Argentina" or "Bolivia" or "Brazil" or "Chile" or "Colombia" or "Ecuador" or "Guyana" or "Paraguay" or "Peru" or "Suriname" or "Uruguay" or "Venezuela" or "Mexico").tw,kf	
10	Combined terms	(1 or 2) and (3 or 4) and (5 or 6) and (7 or 8 or 9)	
11	Population	exp animals/ not humans/	
12	Combined terms	10 not 11	
13	Publication year and languages	limit 12 to (human and English language and yr="2018 -Current")	
14	Publication type	limit 13 to (article or article in press or "preprint (unpublished, non-peer reviewed)" or "review")	1,032

Table S2. Search strategy clinical burden of influenza and influenza-like illness sequelae.

**OVID Embase (database search conducted on 8 September 2023)**			
**#**	**Concept group**	**Search terms**	**Hits**
1	Influenza	exp Influenza/ or exp "Influenza A Virus"/ or exp "Influenza B Virus"/	
2		(Influenza or flu or "influenza like illness" or ILI or influenza-associated or influenza-related or influenza associated or influenza related or ILI-associated or ILI-related or ILI associated or ILI related or influenza like illness associated or influenza like illness related).tw,kf	
3	Complications	exp complications/	
4		(Complication* or sequala* or outcome*).tw,kf	
5	Pulmonary	(pneumonia or superinfection or asthma or COPD or "lower respiratory tract" infection* or "chronic obstructive pulmonary disease" or "acute respiratory distress syndrome" or ARDS).tw,kf	
6	Cardiac	(arrhythmia or cardiac ischemia or myocarditis or myocardial infarction or heart arrest or heart failure or pericardial effusion or pericarditis or pulmonary heart disease).tw,kf	
7	Neurological	(encephalopathy or encephalitis or encephalomyelitis or Guillain Barre syndrome or meningitis or transverse myelitis).tw,kf	
8	Ocular	(conjunctivitis or optic neuritis or retinopathy or uveal effusion).tw,kf	
9	Renal	(acute kidney injury or acute tubular necrosis or glomerulonephritis or hemolytic uremic syndrome or myoglobinuria or rhabdomyolysisacute tubular necrosis).tw,kf	
10	Musculoskeletal	(myopathy or myolysis or myositis).tw,kf	
11	Hepatic	(hepatitis or hepatic vein thrombus or liver disease or portal vein thrombus or transaminitis).tw,kf	
12	Hematologic	(leukopenia or lymphopenia or thrombocytopenia or disseminated intravascular coagulation or embolism or thrombosis or clot or hemolytic uremic syndrome or thrombotic thrombocytopenic purpura or hemophagocytic syndrome).tw,kf	
13	Endocrine	(diabetes or diabetic ketoacidosis).tw,kf	
14	Added complications	(acute coronary disease, fatigue or anxiety or depression or gastric oesophageal reflux disease or hair loss or headache or hyperlipidaemia or hypoxaemia or joint pain or dementia or muscle weakness or skin rash or insomnia or smell disorder or tachycardia or thromboembolism or intracranial haemorrhage or ischaemic stroke or Parkinson's disease or "nerve, nerve root, and plexus disorders" or neuromuscular junction disorders or arrythmias or bradycardia or chest pain or chronic kidney disease or constipation or cough or diarrhoea).tw,kf	
15	Outcomes	exp hospitalization/ or exp mortality/ or exp outpatient/	
16		(case* or hospital* or hospitalization or hospitalisation or mortality or death or ICU or intensive care or critical care or severe illness or severe outcome* or outpatient or mechanical ventilation or supplemental oxygen).tw,kf	
17	Effect measurements	exp incidence/ or exp prevalence/ or epidemiology/ or exp "disease burden"/	
18		(incidence or rate or prevalence or burden or epidemiology or surveillance or disease characteristics).tw,kf	
19	Regions of interests	asia/ or middle east/ or Africa/ or Latin America/ NOT China/ NOT Japan/	
20		(Asia or middle east or Africa or Latin America).tw,kf	
21		("Afghanistan" or "Armenia" or "Azerbaijan" or "Bahrain" or "Bangladesh" or "Bhutan" or "Brunei" or "Cambodia" or "Cyprus" or "Georgia" or "India" or "Indonesia" or "Iran" or "Iraq" or "Israel" or "Jordan" or "Kazakhstan" or "Kuwait" or "Kyrgyzstan" or "Laos" or "Lebanon" or "Malaysia" or "Maldives" or "Mongolia" or "Myanmar" or "Nepal" or "north korea" or "Oman" or "Pakistan" or "Palestine" or "Philippines" or "Qatar" or "Russia" or "saudi arabia" or "Singapore" or "south korea" or "sri lanka" or "Syria" or "Taiwan" or "Tajikistan" or "Thailand" or "Timor-Leste" or "Turkey" or "Turkmenistan" or "united arab emirates" or "UAE" or "Uzbekistan" or "Vietnam" or "Yemen" or "Algeria" or "Angola" or "Benin" or "Botswana" or "burkina faso" or "Burundi" or "Cabo" or "Verde" or "Cameroon" or "central african republic" or "Chad" or "Comoros" or "Congo" or "Democratic Republic of the Congo" or "cote d ivoire" or "Djibouti" or "Egypt" or	

		"equatorial guinea" or "Eritrea" or "Eswatini" or "Ethiopia" or "Gabon" or "Gambia" or "Ghana" or "Guinea" or "Guinea-Bissau" or "Kenya" or "Lesotho" or "Liberia" or "Libya" or "Madagascar" or "Malawi" or "Mali" or "Mauritania" or "Mauritius" or "Morocco" or "Mozambique" or "Namibia" or "Niger" or "Nigeria" or "Rwanda" or "Sao Tome and Principe" or "Senegal" or "Seychelles" or "sierra leone" or "Somalia" or "south africa" or "south sudan" or "Sudan" or "Tanzania" or "Togo" or "Tunisia" or "Uganda" or "Zambia" or "Zimbabwe" or "Argentina" or "Bolivia" or "Brazil" or "Chile" or "Colombia" or "Ecuador" or "Guyana" or "Paraguay" or "Peru" or "Suriname" or "Uruguay" or "Venezuela" or "Mexico").tw,kf	
22		(1 or 2) and (3 or 4 or 5 or 6 or 7 or 8 or 9 or 10 or 11 or 12 or 13 or 14 or 15 or 16) and (17 or 18) and (19 or 20 or 21)	
23	Publication year & population & language	limit 22 to (human and English language and yr="2018 -Current")	
24	Publication type	limit 23 to (article or article in press or "preprint (unpublished, non-peer reviewed)" or "review")	1,648

Table S3. Inclusion and exclusion criteria.

PECO	Inclusion	Exclusion
**Population**	Any human populations, without any restrictions (e.g. age, sex, ethnicity, risk groups)	Studies excluding human populations
**Intervention**	Any	Any
**Comparison**	Any	Any
**Study design**	Intervention studies:<br>• Randomized control trial<br>• Single arm/non-randomized clinical trial<br>Observational studies:<br>• Cohort study<br>• Case-control study<br>• Cross-sectional study<br>• Test-negative study<br>Modelling and simulation study<br>Surveillance reports	SLRs and meta-analyses were screened to identify relevant articles; these were not included in the current SLR
**Outcomes of interest**	Burden of influenza infection:<br>• Influenza infection cases<br>• Outpatient visits due influenza infection<br>• ER visits due influenza infection<br>• Hospitalization due to influenza infection<br>• ICU admission due to influenza infection<br>• Death due to influenza infection<br>Burden of ILI:<br>• ILI cases<br>• Outpatient visits due to ILI<br>• ER visits due to ILI<br>• Hospitalization due to ILI<br>• ICU admission due to ILI<br>• Death due to ILI<br>Burden of influenza sequelae:<br>• Influenza-associated complication outpatient cases<br>• Hospitalization due to influenza-associated complications<br>• ICU admission due to influenza-associated complications<br>• Death due to influenza-associated complications<br>Burden of ILI sequelae:<br>• ILI-associated complication outpatient cases<br>• Hospitalization due to ILI-associated complications<br>• ICU admission due to ILI-associated complications<br>• Death due to ILI-associated complications	Studies not including any of the outcomes of interest
**Effect measurements**	• Prevalence, proportion<br>• Incidence, incidence rate<br>• Case fatality rate<br>• Mortality rate	Studies that did not report any effect measurements for the outcomes specified in the inclusion criteria
**Regions of interest**	Countries in Asia (excluding Mainland China, Hong Kong, Macau, and Japan, including Taiwan), Africa, the Middle East, and Latin America	Studies conducted in China, Japan, and outside of regions of interest
**Language**	English, Chinese, Spanish	Studies not published in English, Chinese or Spanish
**Publication year**	2018 to 8 September 2023	Studies published or data collected before 2018
**Publication type**	Published journal articles, preprints, reports, policy documents from local government agencies and academic institutions, and other grey literature	Conference abstract, case reports/series, commentary and opinion pieces, reviews

ER–emergency room; ICU–intensive care unit; ILI–influenza-like illness; SLR–systematic literature review.

Table S4. Influenza or influenza-like illness associated pulmonary and extra-pulmonary complications.

Sequelae	Complications
Pulmonary sequelae	Pneumonia, superinfection, asthma, lower respiratory tract infection, chronic obstructive pulmonary disease, acute respiratory distress syndrome
Extra-pulmonary sequelae	Arrhythmia, cardiac ischemia, myocarditis, myocardial infarction, heart arrest, heart failure, pericardial effusion, pericarditis, pulmonary heart disease, encephalopathy, encephalitis, encephalomyelitis, Guillain Barre syndrome, meningitis, transverse myelitis, conjunctivitis, optic neuritis, retinopathy, uveal effusion, acute kidney injury, acute tubular necrosis, glomerulonephritis, hemolytic uremic syndrome, myoglobinuria, rhabdomyolysis acute tubular necrosis, myopathy, myolysis, myositis, hepatitis, hepatic vein thrombus, liver disease, portal vein thrombus, transaminitis, leukopenia, lymphopenia, thrombocytopenia, disseminated intravascular coagulation, embolism, thrombosis, clot, hemolytic uremic syndrome, thrombotic thrombocytopenic purpura, hemophagocytic syndrome, diabetes, diabetic ketoacidosis, acute coronary disease, fatigue, anxiety, depression, gastric oesophageal reflux disease, hair loss, headache, hyperlipidaemia, hypoxaemia, joint pain, dementia, muscle weakness, skin rash, insomnia, smell disorder, tachycardia, thromboembolism, intracranial haemorrhage, ischaemic stroke, Parkinson's disease, "nerve, nerve root, and plexus disorders", neuromuscular junction disorders, arrythmias, bradycardia, chest pain, chronic kidney disease, constipation, cough, diarrhoea

Figure S1. Data availability for disease burden of lab-confirmed influenza in emerging markets*.

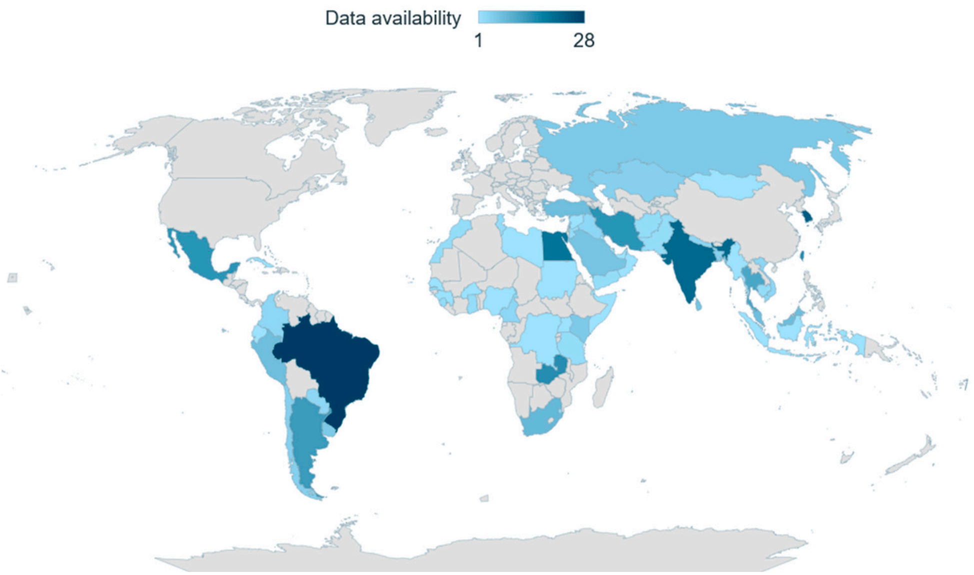

* This systematic review focuses on disease burden in emerging markets, hence excluding studies conducted in China, Japan, Europe, and North America.

Figure S2. Data availability for disease burden of lab-confirmed influenza sequelae in emerging markets*.

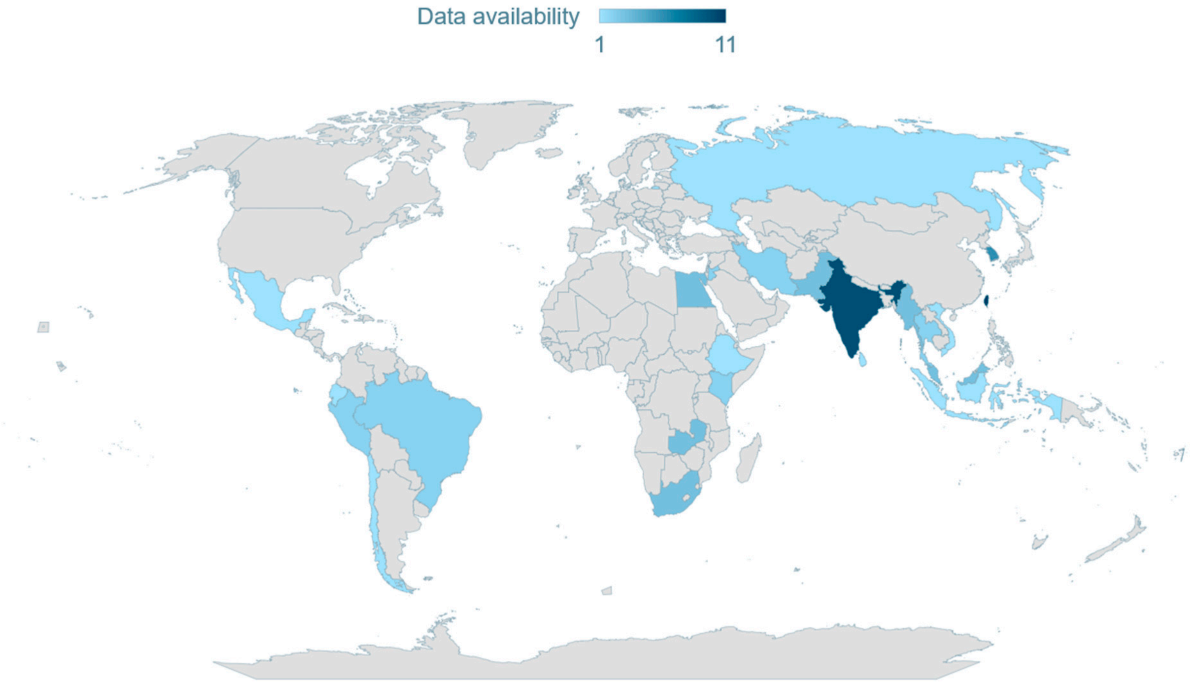

* This systematic review focuses on disease burden in emerging markets, hence excluding studies conducted in China, Japan, Europe, and North America.

Figure S3. Data availability for disease burden of influenza-like-illness in emerging markets*.

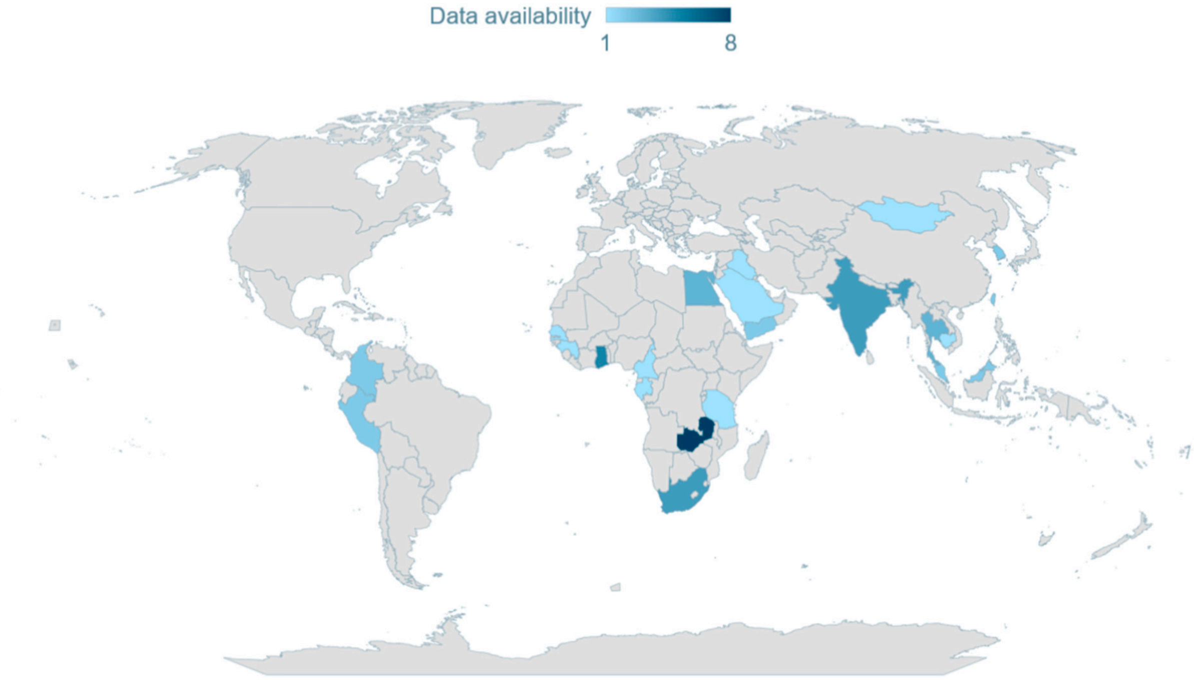

* This systematic review focuses on disease burden in emerging markets, hence excluding studies conducted in China, Japan, Europe, and North America.

Table S5. Data availability by outcomes for disease burden of lab-confirmed influenza infection across four regions.

Influenza infection and associated outcomes	Asia	Sub-Saharan Africa	Latin America	Middle East & North Africa
Influenza infection[1]	43	28	27	17
Influenza outpatient visits[1]	15	9	6	5
Influenza associated ER visits	3	0	0	2
Influenza associated hospitalization	37	12	21	20
Influenza associated ICU admission	11	0	11	6
Influenza associated death	10	2	22	11

[1] Studies spanned multiple regions have been included in the counts for each applicable region category.

Table S6. Data availability by outcomes for disease burden of lab-confirmed influenza sequelae across four regions.

Influenza infection and associated outcomes	Asia	Sub-Saharan Africa	Latin America	Middle East & North Africa
Influenza-associated complication cases/diagnosis	15	4	2	0
Hospitalization due to influenza-associated complications	15	5	2	9
ICU admission due to influenza-associated complications	6	2	0	1
Death/fatality due to influenza-associated complication[1]	12	1	1	1
Outpatient/visits due to influenza-associated complication	2	1	3	1
Emergency room (ER) visits due to influenza-associated complication	0	0	1	0

[1] Studies spanned multiple regions have been included in the counts for each applicable region category.

Table S7. Data availability by outcomes for disease burden of influenza-like illness across four regions.

Influenza infection and associated outcomes	Asia	Sub-Saharan Africa	Latin America	Middle East & North Africa
ILI cases[1]	12	13	2	5
Hospitalization due to ILI/severe acute respiratory infections (SARI)	3	5	1	0
ICU admission due to ILI	1	0	0	0
Death/fatality due to ILI	0	3	1	2
Outpatient/visits due to ILI[1]	4	5	2	2
Emergency room (ER) visits due to ILI	0	0	0	0

[1] Studies spanned multiple regions have been included in the counts for each applicable region category.

Table S8. Overview of study quality.

Lab-confirmed influenza					
**Study quality assessment**	**Total (n=218)**	**Asia (n=89)**	**Sub-Saharan Africa (n=39)**	**Latin America (n=52)**	**Middle East & North Africa (n=38)**
QualSyst scores[1,2]					
Median	89.0%	86.0%	91.0%	91.0%	91.0%
Range (min-max)	29.0-100%	39.3-100%	29.0-100%	54.5-92.5%	55.0-100%
Quality classification[1,2]					
High quality, n (%)	193 (91.0%)	79 (90.8%)	34 (89.5%)	47 (94.0%)	33 (89.2%)
Medium quality, n (%)	16 (7.5%)	6 (6.9%)	3 (7.9%)	3 (6.0%)	4 (10.8%)
Low quality, n (%)	3 (1.4%)	2 (2.3%)	1 (2.6%)	-	-
Not applicable, n	6	2	1	2	1
**Lab-confirmed influenza-associated sequelae**					
**Study quality assessment**	**Total (n=72)**	**Asia (n=39)**	**Sub-Saharan Africa (n=11)**	**Latin America (n=10)**	**Middle East & North Africa (n=12)**
QualSyst scores[1,2]					
Median	82.0%	82.0%	91.0%	84.5%	78.0%
Range (min-max)	29.0-100%	39.3-100%	29.0-95.5%	77.0-95.5%	32.1-100%
Quality classification[1,2]					
High quality, n (%)	53 (81.5%)	25 (78.1%)	8 (72.7%)	10 (100%)	10 (83.3%)
Medium quality, n (%)	7 (10.8%)	4 (12.5%)	2 (18.2%)	-	1 (8.3%)
Low quality, n (%)	5 (7.7%)	3 (9.4%)	1 (9.1%)	-	1 (8.3%)
Not applicable, n	7	7	0	0	0
**Influenza-like illness**					
**Study quality assessment**	**Total (n=45)**	**Asia (n=17)**	**Sub-Saharan Africa (n=17)**	**Latin America (n=4)**	**Middle East & North Africa (n=7)**
QualSyst scores[1,2]					
Median	91.0%	75.0%	90.5%	91.0%	84.0%
Range (min-max)	29.0-100%	29.0-100%	29.0-90.9%	86.0-91.0%	60.7-95.0%
Quality classification[1,2]					
High quality, n (%)	29 (70.7%)	8 (50.0%)	13 (81.2%)	3 (100%)	5 (83.3%)
Medium quality, n (%)	9 (22.0%)	6 (37.5%)	2 (12.5%)	-	1 (16.7%)
Low quality, n (%)	3 (7.3%)	2 (12.5%)	1 (6.3%)	-	-
Not applicable, n	4	1	1	1	1

[1] Studies spanned multiple regions have been included in the counts for each applicable region category; [2] Data extracted directly from surveillance databases were not subjected to quality assessment.

Figure S4. Overview of incidence rate of influenza-like illness cases reported in the studies by region* (n=5 studies).

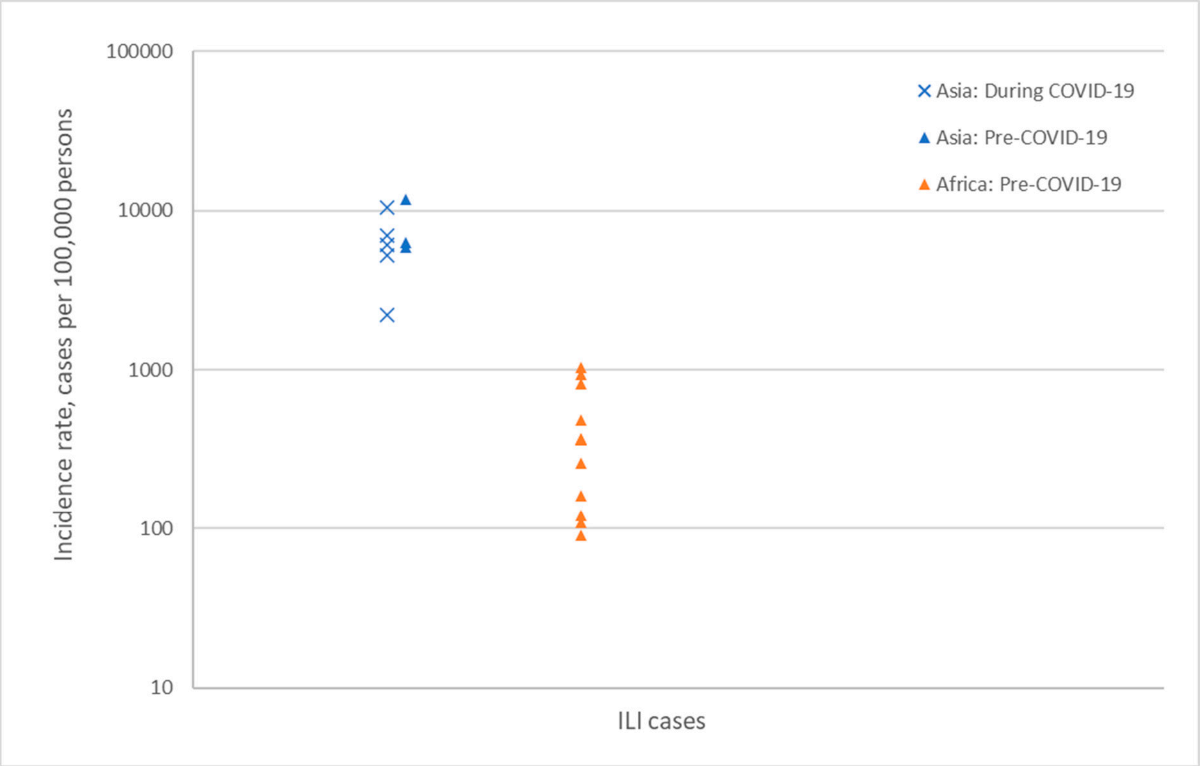

* This figure includes all incidence rates reported in the studies to ensure comprehensiveness of the result presentation. The wide range of incidence rates is due to the various study populations, periods, study designs, and methods used in the included studies.

Figure S5. The median and interquartile range (IQR) of proportions of influenza-like illness cases and its associated outcomes reported A) in all eligible studies, B) in studies that were conducted before COVID-19 pandemic (2018-2019), C) in studies that were conducted during the COVID-19 pandemic (2020-2023) (n=41 studies).

A) Overview of all studies

	**Asia**	**Sub-Saharn Aftica**	**Latin America**	**Middle East & North Africa**
ILI cases	9.9% (14.1%)	10.6% (31.6%)	10.5% (53.3%)	19.3% (56.0%)
Hospitalization associated with ILI	3.72% (-)[1]	9.6% (8.1%)	0.3% (0.3%)	
ICU admission associated with ILI				
Death associated with ILI		0.9% (5.9%)	0.8% (-)[1]	0% (11.3%)
Outpatient visits associated with ILI	11.1% (3.1%)	17.0% (26.2%)	28.8% (5.1%)	43.6% (-)[1]
ER visits associated with ILI				

[1] Only one study. Hence, no interquartile range presented

Number of studies on Proportion of cases, %		
From	To	Color
0		
1	5	
6	10	
11	15	
16	20	
≥21		

B) Pre-COVID-19

	**Asia**	**Sub-Saharn Aftica**	**Latin America**	**Middle East & North Africa**
ILI cases		8.4% (16.4%)	54.8%	
Hospitalization associated with ILI		3.8% (7.0%)	0.3% (0.3%)	
ICU admission associated with ILI				
Death associated with ILI		1.8% (9.0%)	0.8% (-)[1]	22.5% (-)[1]
Outpatient visits associated with ILI	12.4% (1.3%)	17% (0.05%)		
ER visits associated with ILI				

[1] Only one study. Hence, no interquartile range presented

C) During COVID-19

	**Asia**	**Sub-Saharn Aftica**	**Latin America**	**Middle East & North Africa**
ILI cases	20.2% (54.7%)	67.4% (22.3%)		56.7% (12.5%)
Hospitalization associated with ILI	3.7% (-)[1]	9% (-)[1]		
ICU admission associated with ILI				
Death associated with ILI		0% (-)[1]		0% (-)[1]
Outpatient visits associated with ILI	7.5% (-)[1]	25.4% (15.4%)	28.8% (5.1%)	43.6% (-)[1]
ER visits associated with ILI				

[1] Only one study. Hence, no interquartile range presented

Disease burden of lab-confirmed influenza
Incidence rate

Table S9. Summary of range of incidence rate for lab-confirmed influenza infection.

Range (min-max), (number of studies)	Asia		Sub-Saharan Africa		Latin America		Middle East & North Africa	
	Range	Citation	Range	Citation	Range	Citation	Range	Citation
All studies*	540-1278.8 per 100,000 persons (4)	[1-4]	34,100-47,800 per 100,000 persons (1)	[5]	0.7 -112.2 per 100,000 persons (2)	[4,6]	0.1 – 9.55 per 100,00 persons (1)	[7]
Gender								
Female	-	-	-	-	-	-	-	-
Male	-	-	-	-	-	-	-	-
By year								
2018	2.0-6847.0 per 100,000 persons (4)	[1-3,8]	-	-	-	-	-	-
2019	2.0-1278.0 per 100,000 persons (4)	[1-3,8]	-	-	26 per 100,000 persons (2)	[6]	9.55 per 100,000 persons (1)	[7]
2020	54.6-1913.3 per 100,000 persons (4)	[1-3,8]	-	-	7.2 per 100,000 persons (2)	[6]	6 per 100,000 persons (1)	[7]
2021	-	-	-	-	0.9 per 100,000 persons (2)	[6]	0.1 per 100,000 persons (1)	[7]
2022	-	-	-	-	0.7 per 100,000 persons (2)	[6]	-	-
Populations								
Adults >=18	540-684 per 100,000 persons (1)	[4]	-	-	112.2 per 100,000 persons (1)	[4]	-	-
Children and/or adolescents	3940-22,580 per 100,000 persons (1)	[9]	-	-	-	-	-	-
Adults & Children and/or adolescents	0.3-45.9 per 100,000 persons (2)	[1,2]	34,100-47,800 per 100,000 persons (1)	[5]	0.7- 26 per 100,000 persons (1)	[6]	0.1 – 9.55 per 100,00 persons (1)	[7]
Influenza type A								
H1N1pdm09	-	-	-	-	-	-	-	-

H3N4	-	-	-	-	-	-	-	-
H3N2								
Type A not specified			-	-	-	-	-	-
Influenza type B								
B/Yamagata	-	-	-	-	-	-	-	-
B/Victoria	-	-	-	-	-	-	-	-
B/Yamagata & B/Victoria	-	-	-	-	-	-	-	-
Type B not specified	-	-	-	-	-	-	-	-
Other stratification factors	Time period, pregnancy trimester, region	[2,4]	Region	[5]	-	-	-	-

* One study reported number of incident cases without reporting incidence rates [10].

Table S10. Summary of range of incidence rate for hospitalization associated with lab-confirmed influenza infection.

**Range (min-max), (number of studies)**	**Asia**		**Sub-Saharan Africa**		**Latin America**		**Middle East & North Africa**	
	**Range**	**Citation**	**Range**	**Citation**	**Range**	**Citation**	**Range**	**Citation**
All studies	0-9590.0 per 100,000 persons (7)	[4,9,11-15]	-	-	0 - 249 per 100,000 persons (4)	[4,16-18]	See text. (1)	[19]
Gender								
Female	164.6-8,710 per 100,000 persons (2)	[9,15]	-	-	-	-	-	-
Male	98.7-9590 per 100,000 persons (2)	[9,15]	-	-	-	-	-	-
By year								
2018	-	-	-	-	-	-	-	-
2019	-	-	-	-	-	-	-	-
2020	-	-	-	-	-	-	-	-
2021	-	-	-	-	-	-	-	-
2022	-	-						
Populations								
Adults >=18	0-900.0 per 100,000 persons (3)	[4,13,15]	-	-	-	-	-	-

Children and/or adolescents	10,320.0-22,580.0 per 100,000 persons (1)	[9]	-	-	-	-	-	-
Adults & Children and/or adolescents	53.0-9,590.0 per 100,000 persons (3)	[9,11,12]	-	-	2.2- 249 per 100,000 persons (4)	[4,16-18]	-	-
Influenza type A								
H1N1	-	-	-	-	-	-	-	-
H2N3	-	-	-	-	-	-	-	-
Type A not specified	-	-	-	-	-	-	-	-
Influenza type B								
B/Yamagata	-	-	-	-	-	-	-	-
B/Victoria	-	-	-	-	-	-	-	-
B/Yamagata & B/Victoria	-	-	-	-	-	-	-	-
Type B not specified	-	-	-	-	-	-	-	-
Other stratification factor	Season, region, time period	[4,12,20]	-	-	-	-	-	-

Table S11. Summary of range of incidence rate for ICU admission associated with lab-confirmed influenza infection.

Range (min-max), (number of studies)	Asia		Sub-Saharan Africa		Latin America		Middle East & North Africa	
	Range	Citation	Range	Citation	Range	Citation	Range	Citation
All studies	0.8-2.3 per 100,000 persons (1)	[15]	-	-	-	-	-	-
Gender					-	-	-	-
Female	-	-	-	-	-	-	-	-
Male	-	-	-	-	-	-	-	-
By year					-	-	-	-
2018	-	-	-	-	-	-	-	-
2019	-	-	-	-	-	-	-	-
2020	-	-	-	-	-	-	-	-
2021	-	-	-	-	-	-	-	-
2022	-	-	-	-	-	-	-	-
Populations					-	-	-	-
Adults >=18	0.8-2.3 per 100,000 persons (1)	[15]	-	-	-	-	-	-

Children and/or adolescents	-	-	-	-	-	-	-	-
Adults & Children and/or adolescents	-	-	-	-	-	-	-	-
Influenza type A					-	-	-	-
H1N1	-	-	-	-	-	-	-	-
H2N3	-	-	-	-	-	-	-	-
Type A not specified	-	-	-	-	-	-	-	-
Influenza type B					-	-	-	-
B/Yamagata	-	-	-	-	-	-	-	-
B/Victoria	-	-	-	-	-	-	-	-
B/Yamagata & B/Victoria	-	-	-	-	-	-	-	-
Type B not specified	-	-	-	-	-	-	-	-
Other stratification factors	-	-	-	-	-	-	-	-

Table S12. Summary of range of incidence rate for death associated with lab-confirmed influenza infection.

Range (min-max), (number of studies)	Asia		Sub-Saharan Africa		Latin America		Middle East & North Africa	
	Range	Citation	Range	Citation	Range	Citation	Range	Citation
All studies*	0.03-64.0 per 100,000 persons (3)	[3,15,21]	52.7-246.6 per 100,000 persons (1)	[21]	0.3 – 53.9 cases per 100,000 persons (2)	[17,18]	18.8-52.7 cases per 100,000 persons (1)	[21]
Gender								
Female	-	-	-	-	-	-	-	-
Male	-	-	-	-	-	-	-	-
By year								
2018	1.9 per 100,000 persons (1)	[3]	-	-	-	-	-	-
2019	0.7 per 100,00 0persons (1)	[3]	-	-	-	-	-	-
2020	0.5 per 100,000 persons (1)	[3]	-	-	-	-	18.8-52.7 cases per 100,000 persons (1)	[21]
2021	-	-	-	-	-	-	-	-
2022	-	-	-	-	-	-	-	-
Populations								

Adults >=18	0.03-19.2 per 100,000 persons (1)	[15]	-	-	-	-	-	-
Children and/or adolescents	-	-	-	-	-	-	-	-
Adults & Children and/or adolescents	0.1-64.0 per 100,000 person (2)	[3,21]	52.7-246.6 per 100,000 persons (1)	[21]	0.3 – 53.9 cases per 100,000 persons (2)	[17,18]	18.8-52.7 cases per 100,000 persons (1)	[21]
Influenza type A								
H1N1	-	-	-	-	-	-	-	-
H2N3	-	-	-	-	-	-	-	-
Type A not specified	-	-	-	-	-	-	-	-
Influenza type B								
B/Yamagata	-	-	-	-	-	-	-	-
B/Victoria	-	-	-	-	-	-	-	-
B/Yamagata & B/Victoria	-	-	-	-	-	-	-	-
Type B not specified	-	-	-	-	-	-	-	-
Other stratification factors	Region	[21]	Region	[21]	-	-	Region	[21]

* Two studies reported number of incident cases without reporting incidence rates [22,23].

Table S13. Summary of range of incidence rate for outpatient visits associated with lab-confirmed influenza infection.

Range (min-max), (number of studies)	Asia		Sub-Saharan Africa		Latin America		Middle East & North Africa	
	Range	Citation	Range	Citation	Range	Citation	Range	Citation
All studies*	-	-	-	-	20.3 - 12,030 cases per 100,000 persons (2)	[17,18]	-	-
Gender							-	-
Female	-	-	-	-	-	-		
Male	-	-	-	-	-	-	-	-
By year							-	-
2018	-	-	-	-	-	-	-	-
2019	-	-	-	-	-	-	-	-
2020	-	-	-	-	-	-	-	-
2021	-	-	-	-	-	-	-	-
2022	-	-	-	-	-	-	-	-
Populations					-	-	-	-

Adults >=18	-	-	-	-	-	-	-	-
Children and/or adolescents	-	-	-	-	-	-	-	-
Adults & Children and/or adolescents	-	-	-	-	20.3 - 12,030 cases per 100,000 persons (2)	[17,18]		
Influenza type A								
H1N1	-	-	-	-	-	-	-	-
H2N3	-	-	-	-	-	-	-	-
Type A not specified	-	-	-	-	-	-	-	-
Influenza type B								
B/Yamagata	-	-	-	-	-	-	-	-
B/Victoria	-	-	-	-	-	-	-	-
B/Yamagata & B/Victoria	-	-	-	-	-	-	-	-
Type B not specified	-	-	-	-	-	-	-	-
Other stratification factors	-	-	-	-	-	-	-	-

* Four studies reported number of incident cases without reporting incidence rates [23,24,25,26].

Disease burden of lab-confirmed influenza
Proportion of cases

Table S14. Summary of proportion of lab-confirmed influenza cases in the study population.

Range (min-max), (number of studies)	Asia		Sub-Saharan Africa		Latin America		Middle East & North Africa	
	Range	Citation	Range	Citation	Range	Citation	Range	Citation
All studies*	0-77.1% (24)	[3,21,27-48]	0.1-100% (29)	[21,28,39,48-73]	0%-53.7% (13)	[48,74-84]	0.70%-53.5% (12)	[28,48,85-94]
Gender								
Female	30.8-54.6% (2)	[32,35]	1.6-60.3% (6)	[53,55,67,71-73]	49.4% (1)	[74]	40.2%-62.8% (4)	[86,90-92]
Male	28.8-45.4% (2)	[32,35]	0-51.2% (5)	[55,67,71-73]	50.6% (1)	[74]	37.2%-59.8% (4)	[86,90-92]
By year								
2018	3.3-37.8% (8)	[3,32,42,47,48, 93,95,96]	1.12-42.3% (9)	[48,52,54,59-61,63,72]	9.57% (1)	[78]	-	-
2019	2.0-44.4%% (10)	[3,32,33,42,44,47,48,93,95,96]	6.7-44.4% (5)	[48,52,60,63,68]	14.5% (1)	[78]	-	-
2020	0.4-51.4% (8)	[3,33,42,44,48,93,96,97]	0.7-27.5% (3)	[48,52,68]	0.98% (1)	[78]	-	-
2021	0.0-31.3% (4)	[34,48,97,98]	0.0-64.2% (3)	[48,52,68]	3.54% (1)	[99]	-	-
2022	0.3-31.5% (3)	[34,48,97]	0.3-44.3% (1)	[48]	6.8% (1)	[99]	-	-
2023	2.3-34.5% (1)	[48]	0.5-30.8% (1)	[48]	-	-	-	-
Populations								
Adults >=18	0-100% (3)	[4,29,38]	9.0-81.8% (3)	[56,67,69]	6.9% (1)	[75]	-	-
Children and/or adolescents	0.7-24.7% (2)	[28,95]	0-20.0% (3)	[54,56,71]	0%-53.7% (3)	[77,79,99]	-	-
Adults & Children and/or adolescents	0-83.3% (24)	[21,27,30,32-37,39-46,48, 100-104]	0-66.6% (20)	[21,39,50-53,55, 57,59-63, 64,65, 68,70,72,73,105]	0% - 25% (10)	[48,74,76,78,80-84]	0.70% - 53.5% (12)	[28,48,85-94]
Influenza type A								
H1N1	0.0-78.3% (7)	[3,32,36,42,48,97,102]	0-71.4% (9)	[48-50,57,59,63, 70,72,73]	0.15% - 13.61% (2)	[48,75]	0.11%-68.1% (2)	[48,88]

H2N3	1.0-55.7% (7)	[3,32,36,42,97,98,102]	0.3-66.6% (6)	[49,51,57,59,63,70]	36.0% (1)	[82]	-	-
H5	0% (1)	[31]	-	-	-	-	-	-
Type A not specified	0.0-77% (8)	[34,41,43,46,48,96-98]	0.0-64.2% (11)	[48,50-56,62,72,73]	0%-3.64% (2)	[48,77]	0%-100% (2)	[48,85,88,93,94]
H1N1pdm09	-	-	-	-	48.4% (1)	[82]	31.8% (1)	[93]
H1pdm09	-	-	-	-	-	-	100% (1)	[89]
H1	-	-	-	-	-	-	13.5%-43% (2)	[86,87]
H3	-	-	-	-	0% - 7.01% (1)	[48]	0.18%-69.2% (4)	[48,86,87,93]
H5	-	-	-	-	-	-	0% (1)	[89]
Influenza type B								
B/Yamagata	0.0-1.39% (1)	[48]	0.0-70.0% (4)	[48,54,57,63]	0%-33.0% (2)	[48,78]	0%-1.22% (2)	[48,93]
B/Victoria	0.0-15.8% (2)	[48,102]	0.0-39.9% (5)	[48,54,57,63,72]	0.04%-67.0% (2)	[48,78]	0%-3.36% (2)	[48,93]
B/Yamagata & B/Victoria	-	-	-	-	-	-	-	-
Type B not specified	0.0-35.0 (9)	[3,32,34,36,42,43,48,96,97]	0-38.9% (13)	[48-53,55,56,59,62,71-73]	0%-15.5% (3)	[48,77,82]	0%-19.8% (6)	[48,85-88,93]
Other stratification factors	Region, pandemic period, type of illness, type of sample, Charlson Comorbidity Index score, type of insurance, symptoms	[28,32,35,100,102]	Type of illness, region, population type, comorbidity, time period, diagnostic kit, co-worker with history of respiratory illness in the last 12 months, history of respiratory illness in the last 12 months, household	[21,28,39,51,53,62,66-69,73]	SARS-CoV-2 co-infection	[76]	Symptoms, type of work, type of exposure, type of illness, region, vaccination status	[86-90,92]

			member with history of respiratory illness in the last 12 months, number of household members, use of cloth gloves while working					

* Two studies reported number of cases without reporting proportions [106,107].

Table S15. Summary of proportion of hospitalization associated with lab-confirmed influenza infection in the study population.

Range (min-max), (number of studies)	Asia		Sub-Saharan Africa		Latin America		Middle East & North Africa	
	Range	Citation	Range	Citation	Range	Citation	Range	Citation
All studies*	0-98.2% (24)	[3,11,15,30,35,36,105,108-124]	0-61.5% (14)	[53,55,56,58,64,65,122,124-130]	0.13%-94.0% (18)	[6,15,80,99,131-144]	0%-42.2% (17)	[19,87,90,92,122,124,145-155]
Gender								
Female	11.2-62.9% (5)	[15,108,110,115,121]	40.6-100% (1)	[55]	48.9%-52.9% (2)	[134,144]	20.0%-66.7% (4)	[152-155]
Male	33.3-58.3% (5)	[15,108,110,115,121]	0-59.4% (1)	[55]	47.2%-51.1% (2)	[134,144]	33.3%-80.0% (4)	[152-155]
By year								
2018	9.7-33.9% (3)	[3,12,156]	-	-	17.5% (1)	[132]	-	-
2019	10.7-14.8% (2)	[3,156]	-	-	8.6%-13.5% (3)	[132,136,142]	-	-
2020	11.2%	[3]	-	-	0.2% (1)	[136]	-	-
2021	-	-	-	-	-	-	-	-
2022	-	-	-	-	-	-	-	-
Populations								
Adults >=18	0-85.1% (11)	[15,105,109,111-113,118,119,157-159]	-	-	13.7%-94.0% (4)	[131-133,138]	-	-
Children and/or adolescents	0-40.6% (9)	[3,11,30,108,115,121,160-162]	-	-	2.52%-5.28% (3)	[99,140,142]	0%-16.3% (5)	[146-148,150,155]

Adults & Children and/or adolescents	0-94.9% (12)	[11,30,35,36,110,115,117,121,122,124,156,163]	0-61.5% (14)	[53,55,56,58,64,65,122,124-130]	0.66% - 61.5% (8)	[6,15,80,134,136,137,143,144]	1.5% - 42.2% (12)	[19,87,90,92,122,124,145,149,151-154]
Influenza type A								
H1N1	1.9-49.2% (4)	[98,110,112,121]	0-61.5% (3)	[124,127,130]	35.1% - 64.4% (3)	[15,137,144]	14.3% - 36.6% (2)	[145,149]
H1N1pdm09	-	-	-	-	-	-	18.7%-87.0% (2)	[90,147]
H1	-	-	-	-	-	-	0% (1)	[87]
H2N3	6.7-67.3% (1)	[110]	0-18.1% (3)	[56,124,130]	-	-	-	-
H3N2	-	-	-	-	34.5%-37.1% (2)	[15,144]	4.3% - 13.0% (2)	[145,149]
H3	-	-	-	-	-	-	41.7% - 44.4% (2)	[87,90]
Type A not specified	26.0-85.1% (2)	[11,105]	0-59.4% (6)	[53,55,58,124,127,128]	5.24% (1)	[139]	15.1% (1)	[149]
Influenza type B								
B/Yamagata	-	-	0 (2)	[124,130]	-	-	-	-
B/Victoria	-	-	0-2.8% (2)	[124,130]	-	-	-	-
B/Yamagata & B/Victoria	-	-	-	-	-	-	-	-
Type B not specified	12.0-37.0% (3)	[11,105,108]	0-100%	[53,55,56,58,124,125,127,128]	1.15% - 1.40% (3)	[15,139,144]	0% - 55.6% (4)	[87,90,147,149]
Other stratification factor	Surveillance type, region	[116,124]	Region	[124]	Comorbidities, Disease outcome, Education level, ICU admission, Influenza season, Living in flu outbreak region, ventilatory support, place of residence, postpartum women, region, signs and symptoms, use	[6,134,135,137,139,141,144]	-	-

					of anti-viral, vaccination status			

* Three studies reported number of cases without reporting proportions [141,164,165].

Table S16. Summary of proportion of ICU admission associated with lab-confirmed influenza infection in the study population.

**Range (min-max), (number of studies)**	**Asia**		**Sub-Saharan Africa**		**Latin America**		**Middle East & North Africa**	
	**Range**	**Citation**	**Range**	**Citation**	**Range**	**Citation**	**Range**	**Citation**
All studies*	0.6-69.4% (10)	[115,116,166-173]	5.8-33.3% (1)	[166]	0%-39.2% (11)	[80,99,134,166, 170,174-179]	2.7% - 30.0% (5)	[87,90,145,166, 180]
Gender								
Female	-	-	-	-	58.9% (1)	[178]	-	-
Male	-	-	-	-	41.1% (1)	[178]	-	-
By year								
2018	-	-	-	-	1.97% (1)	[177]	-	-
2019	-	-	-	-	3.06% (1)	[177]	-	-
2020	-	-	-	-	-	-	-	-
2021	-	-	-	-	-	-	-	-
2022	-	-	-	-	-	-	-	-
Populations								
Adults >=18	0-69.4% (10)	[115,116,166-173]	-	-	23%-28.3% (2)	[175,176]	-	-
Children and/or adolescents	0.8-42.0% (2)	[161,169]	-	-	0%-6.06% (2)	[99,179]	2.8% (1)	[180]
Adults & Children and/or adolescents	0-56.6% (5)	[115,116,166,172,173]	5.8-33.3% (1)	[166]	3.7%-39.2% (5)	[80,134,170,174,177,178]	2.7% - 30.0% (4)	[87,90,145,166]
Influenza type A								
H1N1	0-32.0% (2)	[171,173]	-	-	57.1% (1)	[178]	6.8% (1)	[181]
H2N3	-	-	-	-	41.0% (1)	[178]		
H3N2					-	-	10.8% (1)	[181]
H3					-	-	26.7% (1)	[87]

Type A not specified	11.5-19.2% (1)	[116]	-	-	-	-	-	-
Influenza type B								
B/Yamagata	-	-	-	-	-	-	-	-
B/Victoria	-	-	-	-	-	-	6.9% (1)	[181]
B/Yamagata & B/Victoria	-	-	-	-	-	-	-	-
Type B not specified	-	-	-	-	1.79% (1)	[178]	40.04% (1)	[87]
Other stratification factor	Type of patients, ICU admission duration, region	[159,166,171]	Region	[166]	Signs & Symptoms, Comorbidities, Vaccination status, Type of ventilation, Region	[166,178]	-	-

* One study reported number of cases without reporting proportions [182].

Table S17. Summary of proportion of death associated with lab-confirmed influenza infection in the study population.

Range (min-max), (number of studies)	Asia		Sub-Saharan Africa		Latin America		Middle East & North Africa	
	Range	Citation	Range	Citation	Range	Citation	Range	Citation
All studies*	0-47.0% (12)	[110,115,116,158, 159,166-171,173]	0-3.7% (3)	[55,64,166]	0% - 18.1% (9)	[74,80,134,137, 170,174,178,183,184]	0% - 35.4% (11)	[87,90,145,151,152,166,180,181,185-187]
Gender								
Female	45.5% (1)	[115]	-	-	17.6% - 54.3% (5)	[174-176,183,188]	34.6%-39.1% (2)	[152,185]
Male	54.5% (1)	[115]	-	-	18.1% - 68.7% (5)	[174-176,183,188]	60.9%-65.4% (2)	[152,185]
By year								
2018	0.1% (1)	[169]	-	-	-	-	-	-
2019	0.04% (1)	[169]	-	-	-	-	-	-
2020	0.1% (1)	[169]	-	-	3.06% - 10.2% (2)	[176,189]	-	-
2021	-	-	-	-	1.01% - 26.3% (2)	[176,189]	-	-
2022	-	-	-	-	0.28% - 63.5% (2)	[176,189]	-	-
Populations								

Adults >=18	0-81.8% (4)	[159,167,168,171]	-	-	15.1%- 24.6% (4)	[175,176,188,190]	-	-
Children and/or adolescents	-	-	-	-	-	-	0% (1)	[180]
Adults & Children and/or adolescents	0-54.5% (5)	[110,115,116,166,173]	0-3.7% (3)	[55,64,166]	0% - 18.1% (9)	[74,80,134,137,170,174,178,183,184]	0.9% -35.4%	[87,90,145,151,152,166,181,185-187]
Influenza type A								
H1N1	0-100% (3)	[168,171,173]	-	-	86.9%-3.4% (4)	[174-176,178]	-	-
H1N1pdm09	-	-	-	-	-	-	3.9% (1)	[181]
H2N3	-	-	-	-				
H3N2	-	-	-	-	7.1% - 59.8% (3)	[174-176]	2.4% (1)	[181]
H1	-	-	-	-	-	-	0% (1)	[87]
H3	-	-	-	-	-	-	20% (1)	[87]
Type A not specified	7.7-17.6% (1)	[116]	0% (2)	[55,64]	29.2% - 82.6% (2)	[137,175]	-	-
Influenza type B								
B/Yamagata	-	-	-	-	0.1% - 4.0% (2)	[174,176]	-	-
B/Victoria	-	-	-	-	1.5% (1)	[174]	1.4% (1)	[181]
B/Yamagata & B/Victoria	-	-	-	-	-	-	-	-
Type B not specified	-	-	0% (2)	612, 2442	0% - 5.2% (3)	[174,175,178]	20.0%	[87]
Other stratification factors	Comorbidity, symptoms, type of death, region	[158,166,169,171]	Region	G5	Comorbidities, Hospitalization, ICU admission, Influenza season, Medical history, Patient type (Ambulatory/ Inpatient) , Pneumococcal	[137,166,174-176,178,184,188,189]	Underlying disease	[185]

					vaccination status, Place of occurrence, Region/ Respiratory / Ventilatory support, Symptoms, Vaccination status			

* Two studies reported number of cases without reporting proportions [191,192].

Table S18. Summary of proportion of outpatient visits associated with lab-confirmed influenza infection in the study population.

Range (min-max), (number of studies)	Asia		Sub-Saharan Africa		Latin America		Middle East & North Africa	
	Range	Citation	Range	Citation	Range	Citation	Range	Citation
All studies*	0-90.4% (7)	[3,20,35,36,98,193,194]	0-81.0% (8)	[55,65,73,125,195-198]	0.46% - 23.6% (4)	[137,193,199,200]	2.3% - 61.5% (5)	[87,90,92,153,201]
Gender								
Female	-	-	1.2-16.2% (2)	[197,198]	51.2% (1)	[200]	63.4% (1)	[201]
Male	-	-	0.6-17.5% (2)	[197,198]	48.8% (1)	[200]	36.6% (1)	[201]
By year								
2018	7.2-90.3% (2)	[3,193]	-	-	-	-	-	-
2019	89.3% (1)	[3]	-	-	-	-	-	-
2020	88.7% (1)	[3]	-	-	-	-	-	-
2021	-	-	-	-	-	-	-	-
2022	-	-	-	-	-	-	-	-
Populations								
Adults >=18	0-68.1% (2)	[98,194]	-	-	12.0% (1)	[200]	-	-
Children and/or adolescents	0-3.6% (1)	[202]	1.4-7.0% (1)	[196]	12.7% (1)	[199]	25.3% (1)	[201]
Adults & Children and/or adolescents	0-90.4% (4)	[20,35,36,193]	0-81.0% (7)	[55,65,73,125,195,197,198]	0.46% - 7.4% (2)	[137,193]	2.3% - 61.5% (4)	[87,90,92,153]
Influenza type A								

H1N1	0 (1)	[194]	0-70.6% (2)	[125,198]	12.0% -23.6% (2)	[137,199]	9.6% (1)	[201]
H2N3	68.1% (1)	Cc [98]	17.6% (1)	[198]	11.4% - 41.0% (2)	[137,199]		
H3N2	-	-	-	-	-	-	15.3% (1)	[201]
H3	-	-	-	-	-	-	59.5% (1)	[90]
H1pdm09	-	-	-	-	-	-	12.7% (1)	[90]
Type A not specified	5.2% (1)	[194]	0.9-11.8% (5)	[55,73,125,196,197]	1.39% (1)	[137]	-	-
Influenza type B								
B/Yamagata	-	-	-	-	2.79% (1)	[137]	-	-
B/Victoria	-	-	-	-	0.46% (1)	[137]	-	-
B/Yamagata & B/Victoria	-	-	-	-	-	-	-	-
Type B not specified	6.6% (1)	[194]	0-11.8% (5)	[55,73,125,196,197]			0.4% – 27.3% (2)	[90,201]
Other stratification factors	-	-	Region, type of patients	[73,125]	Region, Database, Comorbidities, Clinical Characteristics	[199,200]	-	-

*One study reported number of cases without reporting proportions [203].

Table S19. Summary of proportion of emergency room visits associated with lab-confirmed influenza infection in the study population.

Range (min-max), (number of studies)	Asia		Sub-Saharan Africa		Latin America		Middle East & North Africa	
	Range	Citation	Range	Citation	Range	Citation	Range	Citation
All studies	0.1-7.3% (1)	[204]	-	-	-	-	1.2% - 17.9% (2)	[205,206]
Gender					-	-	-	-
Female	-	-	-	-	-	-	-	-
Male	-	-	-	-	-	-	-	-
By year					-	-	-	-
2018	-	-	-	-	-	-	-	-

2019	-	-	-	-	-	-	-	-
2020	-	-	-	-	-	-	-	-
2021	-	-	-	-	-	-	-	-
2022	-	-	-	-	-	-	-	-
Populations					-	-	-	-
Adults >=18	-	-	-	-	-	-	-	-
Children and/or adolescents	-	-	-	-	-	-	-	-
Adults & Children and/or adolescents	0.1-7.3% (1)	[204]	-	-	-	-	-	-
Influenza type A					-	-	-	-
H1N1	-	-	-	-	-	-	-	-
H2N3	-	-	-	-	-	-	-	-
Type A not specified	-	-	-	-	-	-	10.9%-17.9%	[205,206]
Influenza type B					-	-	-	-
B/Yamagata	-	-	-	-	-	-	-	-
B/Victoria	-	-	-	-	-	-	-	-
B/Yamagata & B/Victoria	-	-	-	-	-	-	5.1% - 21.4%	[205,206]
Type B not specified	-	-	-	-			1.2%	[206]
Other stratification factors	Season	[204]	-	-	-	-	SARS-CoV-2 co-infection	[206]

Disease burden of lab-confirmed influenza-associated sequelae

Incidence rate

Table S20. Summary of range of incidence rate for lab-confirmed influenza-associated sequelae cases.

**Acute sequelae**								
**Range (min-max), (number of studies)**	**Asia**		**Sub-Saharan Africa**		**Latin America**		**Middle East & North Africa**	
	**Range**	**Citation**	**Range**	**Citation**	**Range**	**Citation**	**Range**	**Citation**
Overall	0.2-30.5 per 100,000 persons (3)	[207-209]	-	-	-	-	-	-
Gender								
Female	-	-	-	-	-	-	-	-
Male	-	-	-	-	-	-	-	-
By year								
2018	-	-	-	-	-	-	-	-
2019	-	-	-	-	-	-	-	-
2020	-	-	-	-	-	-	-	-
2021	-	-	-	-	-	-	-	-
2022	-	-	-	-	-	-	-	-
Populations								
Adults >=18	0.2-30.5 per 100,000 persons (3)	[207-209]	-	-	-	-	-	-
Children and/or adolescents	0.7-6.0 per 100,000 persons (3)	[207-209]	-	-	-	-	-	-
Adults & Children and/or adolescents	0.2-30.5 per 100,000 persons (3)	[207-209]	-	-	-	-	-	-
Influenza type A								
H1N1	-	-	-	-	-	-	-	-
H2N3	-	-	-	-	-	-	-	-
H3N2	-	-	-	-	-	-	-	-
Type A not specified	-	-	-	-	-	-	-	-
Influenza type B								

B/Yamagata	-	-	-	-	-	-	-	-
B/Victoria	-	-	-	-	-	-	-	-
B/Yamagata & B/Victoria	-	-	-	-	-	-	-	-
Type B not specified	-	-	-	-	-	-	-	-
Other stratification factors	-	-	-	-	-	-	-	-
**Long-term sequelae**								
**Range (min-max), (number of studies)**	**Asia**		**Sub-Saharan Africa**		**Latin America**		**Middle East & North Africa**	
	**Citation**	**Range**	**Citation**	**Range**	**Citation**	**Range**	**Citation**	**Range**
Overall	7.7-197.8 per 100,000 persons (1)	[35]	-	-	-	-	-	-
Gender								
Female	-	-	-	-	-	-	-	-
Male	-	-	-	-	-	-	-	-
By year								
2018	-	-	-	-	-	-	-	-
2019	-	-	-	-	-	-	-	-
2020	-	-	-	-	-	-	-	-
2021	-	-	-	-	-	-	-	-
2022	-	-	-	-	-	-	-	-
Populations								
Adults >=18	-	-	-	-	-	-	-	-
Children and/or adolescents	-	-	-	-	-	-	-	-
Adults & Children and/or adolescents	7.7-197.8 per 100,000 persons (1)	[35]	-	-	-	-	-	-
Influenza type A								
H1N1	-	-	-	-	-	-	-	-
H2N3	-	-	-	-	-	-	-	-
Type A not specified	-	-	-	-	-	-	-	-
Influenza type B								
B/Yamagata	-	-	-	-	-	-	-	-
B/Victoria	-	-	-	-	-	-	-	-
B/Yamagata & B/Victoria	-	-	-	-	-	-	-	-

Type B not specified	-	-	-	-	-	-	-	-
Other stratification factors	-	-	-	-	-	-	-	-

Table S21. Summary of range of incidence rate for hospitalization due to lab-confirmed influenza-associated sequelae.

**Acute sequelae**								
**Range (min-max), (number of studies)**	**Asia**		**Sub-Saharan Africa**		**Latin America**		**Middle East & North Africa**	
	**Range**	**Citation**	**Range**	**Citation**	**Range**	**Citation**	**Range**	**Citation**
Overall	0.1-3.8 per 100,000 persons (1)	[170]	0.0-176.0 (1)	[210]	2.2-20.3 per 100,000 persons (1)	[211]	-	-
Gender								
Female	-	-	-	-	-	-	-	-
Male	-	-	-	-	-	-	-	-
By year								
2018	-	-	5-176 per 100,000 persons (1)	[210]	-	-	-	-
2019	-	-	3-68 per 100,000 persons (1)	[210]	-	-	-	-
2020	-	-	0-8 per 100,000 persons (1)	[210]	-	-	-	-
2021	-	-	18-74 per 100,000 persons (1)	[210]	-	-	-	-
2022	-	-	22-138 per 100,000 persons (1)	[210]	2.2-20.3 per 100,000 persons (1)	[211]	-	-
Populations								
Adults >=18	-	-	-	-	2.2-11.2 per 100,000 persons (1)	[211]	-	-
Children and/or adolescents	-	-	0-176 per 100,000 persons (1)	[210]	2.4-20.3 per 100,000 persons (1)	[211]	-	-
Adults & Children and/or adolescents	-	-	-	-	2.2-20.3 per 100,000 persons (1)	[211]	-	-

Influenza type A								
H1N1	-	-	-	-	-	-	-	-
H2N3	-	-	-	-	-	-	-	-
Type A not specified	-	-	5-132 per 100,000 persons (1)	[210]	-	-	-	-
Influenza type B								
B/Yamagata	-	-	-	-	-	-	-	-
B/Victoria	-	-	-	-	-	-	-	-
B/Yamagata & B/Victoria	-	-	-	-	-	-	-	-
Type B not specified	-	-	0-53 per 100,000 persons (1)	[210]	-	-	-	-
Other stratification factors	-	-	-	-	-	-	-	-
**Long-term sequelae**								
No studies reported data on long-term sequelae.								

Table S22. Summary of range of incidence rate for death due to lab-confirmed influenza-associated sequelae.

**Acute sequelae**								
**Range (min-max), (number of studies)**	**Asia**		**Sub-Saharan Africa**		**Latin America**		**Middle East & North Africa**	
	**Range**	**Citation**	**Range**	**Citation**	**Range**	**Citation**	**Range**	**Citation**
Overall	0.0-4.3 per 100,000 persons (4)	[207-209,212]	-	-	-	-	-	-
Gender								
Female	-	-	-	-	-	-	-	-
Male	-	-	-	-	-	-	-	-
By year								
2018	0.04-0.2 per 100,000 persons (1)	[212]	-	-	-	-	-	-
2019	0.1-0.2 per 100,000 persons (1)	[212]	-	-	-	-	-	-
2020	-	-	-	-	-	-	-	-
2021	-	-	-	-	-	-	-	-
2022	-	-	-	-	-	-	-	-

Populations								
Adults >=18	0.0-4.3 per 100,000 persons (3)	[207-209]	-	-	-	-	-	-
Children and/or adolescents	0.0-1.3 per 100,000 persons (4)	[207-209,212]	-	-	-	-	-	-
Adults & Children and/or adolescents	0.0-4.3 per 100,000 persons (4)	[207-209,212]	-	-	-	-	-	-
Influenza type A								
H1N1	-	-	-	-	-	-	-	-
H2N3	-	-	-	-	-	-	-	-
Type A not specified	-	-	-	-	-	-	-	-
Influenza type B								
B/Yamagata	-	-	-	-	-	-	-	-
B/Victoria	-	-	-	-	-	-	-	-
B/Yamagata & B/Victoria	-	-	-	-	-	-	-	-
Type B not specified	-	-	-	-	-	-	-	-
Other stratification factors	-	-	-	-	-	-	-	-
**Long-term sequelae**								
No studies reported data on long-term sequelae.								

Table S23. Summary of range of incidence rate for outpatient visits due to lab-confirmed influenza-associated sequelae.

**Acute sequelae**								
**Range (min-max), (number of studies)**	**Asia**		**Sub-Saharan Africa**		**Latin America**		**Middle East & North Africa**	
	**Range**	**Citation**	**Range**	**Citation**	**Range**	**Citation**	**Range**	**Citation**
Overall	-	-	0.21 pe 100,000 persons (1)	[24]	-	-	-	-
Gender								
Female	-	-	-	-	-	-	-	-
Male	-	-	-	-	-	-	-	-
By year								
2018	-	-	0.21 per 100,000 persons (1)	[24]	-	-	-	-

2019	-	-	-	-	-	-	-	-
2020	-	-	-	-	-	-	-	-
2021	-	-	-	-	-	-	-	-
2022	-	-	-	-	-	-	-	-
Populations								
Adults >=18	-	-	-	-	-	-	-	-
Children and/or adolescents	-	-	-	-	-	-	-	-
Adults & Children and/or adolescents	-	-	-	-	-	-	-	-
Influenza type A								
H1N1	-	-	-	-	-	-	-	-
H2N3	-	-	-	-	-	-	-	-
Type A not specified	-	-	-	-	-	-	-	-
Influenza type B								
B/Yamagata	-	-	-	-	-	-	-	-
B/Victoria	-	-	-	-	-	-	-	-
B/Yamagata & B/Victoria	-	-	-	-	-	-	-	-
Type B not specified	-	-	-	-	-	-	-	-
Other stratification factors								
**Long-term sequelae**								
No studies reported data on long-term sequelae.								

Disease burden of lab-confirmed influenza-associated sequelae

Proportion of cases

Table S24. Summary of proportion of lab-confirmed influenza-associated sequelae cases in the study population.

**Acute sequelae**								
**Range (min-max), (number of studies)**	**Asia**		**Sub-Saharan Africa**		**Latin America**		**Middle East & North Africa**	
	**Range**	**Citation**	**Range**	**Citation**	**Range**	**Citation**	**Range**	**Citation**
Overall	0.0-100% (6)	[30,102,207-209,213]	0.0-38.0% (2)	[214,215]	0.0-0.3% (1)	[216]	-	-
Gender								
Female	-	-	-	-	-	-	-	-
Male	-	-	-	-	-	-	-	-
By year								
2018	-	-	32% (1)	[214]	-	-	-	-
2019	-	-	-	-	-	-	-	-
2020	0.0-27.3% (1)	[30]	-	-	-	-	-	-
2021	-	-	-	-	-	-	-	-
2022	-	-	-	-	-	-	-	-
Populations								
Adults >=18	-	-	-	-	-	-	-	-
Children and/or adolescents	-	-	-	-	-	-	-	-
Adults & Children and/or adolescents	0.0-75.0% (2)	[30,213]	0-38% (2)	[214,215]	0-0.3% (1)	[216]	-	-
Influenza type A								
H1N1	-	-	32% (1)	[214]	-	-	-	-
H2N3	-	-	-	-	-	-	-	-
Type A not specified	-	-	7-38% (1)	[215]	0-0.2% (1)	[216]	-	-
Influenza type B								
B/Yamagata	-	-	-	-	-	-	-	-
B/Victoria	-	-	-	-	-	-	-	-
B/Yamagata & B/Victoria	-	-	-	-	-	-	-	-
Type B not specified	-	-	0-14% (1)	[215]	0.2-0.3% (1)	[216]	-	-
Other stratification factors	Gene mutations, type of illness	[102,213]	-	-	Virological test technique (1)	[216]	-	-

Long-term sequelae								
Range (min-max), (number of studies)	Asia		Sub-Saharan Africa		Latin America		Middle East & North Africa	
	Citation	Range	Citation	Range	Citation	Range	Citation	Range
Overall	0.2-48.9% (1)	[35]	-	-	-	-	-	-
Gender								
Female	-	-	-	-	-	-	-	-
Male	-	-	-	-	-	-	-	-
By year								
2018	-	-	-	-	-	-	-	-
2019	-	-	-	-	-	-	-	-
2020	-	-	-	-	-	-	-	-
2021	-	-	-	-	-	-	-	-
2022	-	-	-	-	-	-	-	-
Populations								
Adults >=18	-	-	-	-	-	-	-	-
Children and/or adolescents	-	-	-	-	-	-	-	-
Adults & Children and/or adolescents	0.2-16.4% (1)	[35]	-	-	-	-	-	-
Influenza type A								
H1N1	-	-	-	-	-	-	-	-
H2N3	-	-	-	-	-	-	-	-
Type A not specified	-	-	-	-	-	-	-	-
Influenza type B								
B/Yamagata	-	-	-	-	-	-	-	-
B/Victoria	-	-	-	-	-	-	-	-
B/Yamagata & B/Victoria	-	-	-	-	-	-	-	-
Type B not specified	-	-	-	-	-	-	-	-
Other stratification factors	Time from diagnosis	[35]	-	-	-	-	-	-

Table S25. Summary of proportion of hospitalization due to lab-confirmed influenza-associated sequelae in the study population.

Acute sequelae*								
Range (min-max), (number of studies)	Asia		Sub-Saharan Africa		Latin America		Middle East & North Africa	
	Range	Citation	Range	Citation	Range	Citation	Range	Citation
Overall	0.0-100.0% (11)	[108,170,173,217-224]	0.8-27.0% (4)	[126,215,225,226]	3.6% - 10.6%	[6,143]	0.0-100.0% (9)	[87,90,180,181,186,187,227-229]

					(2)			
Gender								
Female	0.48-54.7% (2)	[217,223]	-	-	-	-	36.0% (1)	[229]
Male	0.48-45.2% (2)	[217,223]	-	-	-	-	64.0% (1)	[229]
By year								
2018	-	-	-	-	-	-	21.25%	[186]
2019	31.3-50.2% (1)	[219]	-	-	-	-	-	-
2020	-	-	0.8% (1)	[126]	-	-	-	-
2021	-	-	-	-	-	-	-	-
2022	5.6-54.7% (1)	[217]	-	-	-	-	-	-
Populations								
Adults >=18	7.5-37.7% (1)	[217]	-	-	6.9% (1)	[143]	8.0-78.0% (1)	[181]
Children and/or adolescents	5.6-16.9% (1)	[217]	-	-	3.6% (1)	[143]	1.0-100% (3)	[181,227,229]
Adults & Children and/or adolescents	5.6-37.7% (1)	[217]	-	-	-	-	1.0-100% (3)	[181,227,229]
Influenza type A								
H1N1	5.6-54.7% (1)	[217]	-	-	-	-	0.0-10.5% (2)	[181,229]
H2N3			-	-	-	-	0.0-26.5% (2)	[181,229]
Type A not specified	-	-	-	-	-	-	11.0% (1)	[229]
Influenza type B								
B/Yamagata	-	-	-	-	-	-	-	-
B/Victoria	-	-	-	-	-	-	0.0-18.8% (1)	[181]
B/Yamagata & B/Victoria	-	-	-	-	-	-	-	-
Type B not specified	-	-	-	-	-	-	1.3% (1)	[229]
Other stratification factors	-	-	-	-	-	-	Antiviral therapy, coinfection	[87,229]
**Long-term sequelae**								
No studies reported data on long-term sequelae.								

* Two studies reported number of cases without reporting proportions [230,231].

Table S26. Summary of proportion of ICU admission due to lab-confirmed influenza-associated sequelae in the study population.

**Acute sequelae**								
**Range (min-max), (number of studies)**	**Asia**		**Sub-Saharan Africa**		**Latin America**		**Middle East & North Africa**	
	**Range**	**Citation**	**Range**	**Citation**	**Range**	**Citation**	**Range**	**Citation**
Overall	2.0-100.0% (5)	[167,173,218,232,233]	11.0-61.0% (2)	[215,225]	-	-	28.0-40.0% (1)	[229]
Gender								
Female	-	-	-	-	-	-	-	-
Male	-	-	-	-	-	-	-	-
By year								
2018	-	-	-	-	-	-	-	-
2019	-	-	-	-	-	-	-	-
2020	-	-	-	-	-	-	-	-
2021	-	-	-	-	-	-	-	-
2022	-	-	-	-	-	-	-	-
Populations								
Adults >=18	4.4-77.8% (5)	[25,167,173,218,233]	-	-	-	-	-	-
Children and/or adolescents	2.0-100.0% (1)	[232]	-	-	-	-	28.0-40.0% (1)	[229]
Adults & Children and/or adolescents	2.0-100.0% (6)	[25,167,173,218,232,233]	16.0-61.0% (2)	[215,225]	-	-	-	-
Influenza type A								
H1N1	18.2-55.6% (3)	[173,218,233]	-	-	-	-	-	-
H2N3	-	-	-	-	-	-	-	-
Type A not specified	13.7-13.9% (2)	[173,232]	16.0% (1)	[215]	-	-	-	-
Influenza type B								
B/Yamagata	-	-	-	-	-	-	-	-
B/Victoria	-	-	-	-	-	-	-	-
B/Yamagata & B/Victoria	-	-	-	-	-	-	-	-
Type B not specified	2.0% (1)	[232]	-	-	-	-	-	-
Other stratification factors	Level of care, type of infection, ventilation	[218,232]	-	-	-	-	-	-
**Long-term sequelae**								
No studies reported data on long-term sequelae.								

Table S27. Summary of proportion of death due to lab-confirmed influenza-associated sequelae in the study population.

**Acute sequelae**								
**Range (min-max), (number of studies)**	**Asia**		**Sub-Saharan Africa**		**Latin America**		**Middle East & North Africa**	
	**Range**	**Citation**	**Range**	**Citation**	**Range**	**Citation**	**Range**	**Citation**
Overall	0.0-100.0% (6)	[169,173,207-209,218]	-	-	-	-	5.9-25.0% (1)	[228]
Gender								
Female	-	-	-	-	-	-	-	-
Male	-	-	-	-	-	-	-	-
By year								
2018	51.5% (1)	[169]	-	-	-	-	-	-
2019	51.3% (1)	[169]	-	-	-	-	-	-
2020	45.6% (1)	[169]	-	-	-	-	-	-
2021	-	-	-	-	-	-	-	-
2022	-	-	-	-	-	-	-	-
Populations								
Adults >=18	0.0-64.1% (3)	[207-209]	-	-	-	-	-	-
Children and/or adolescents	0.0-2.1% (3)	[207-209]	-	-	-	-	-	-
Adults & Children and/or adolescents	0.0-64.1% (3)	[207-209]	-	-	-	-	5.9-25.0% (1)	[228]
Influenza type A								
H1N1	18.2-75.0% (2)	[173,209]	-	-	-	-	-	-
H2N3	21.8% (1)	[209]	-	-	-	-	-	-
Type A not specified	11.1% (1)	[218]	-	-	-	-	-	-
Influenza type B								
B/Yamagata	-	-	-	-	-	-	-	-
B/Victoria	-	-	-	-	-	-	-	-
B/Yamagata & B/Victoria	-	-	-	-	-	-	-	-
Type B not specified	1.3% (1)	[209]	-	-	-	-	-	-
Other stratification factors	-	-	-	-	-	-	Secondary bacterial infection (1)	[228]
**Long-term sequelae**								
No studies reported data on long-term sequelae.								

Table S28. Summary of proportion of outpatient visits due to lab-confirmed influenza-associated sequelae in the study population.

**Acute sequelae***								
**Range (min-max), (number of studies)**	**Asia**		**Sub-Saharan Africa**		**Latin America**		**Middle East & North Africa**	
	**Range**	**Citation**	**Range**	**Citation**	**Range**	**Citation**	**Range**	**Citation**
Overall	-	-	0% (1)	[24]	0.0-75.6% (2)	[199,234]	1.0-100.0% (1)	[235]
Gender								
Female	-	-	-	-	-	-	-	-
Male	-	-	-	-	-	-	-	-
By year								
2018	-	-	0% (1)	[24]	-	-	-	-
2019	-	-	-	-	-	-	-	-
2020	-	-	-	-	-	-	-	-
2021	-	-	-	-	-	-	-	-
2022	-	-	-	-	12.2-75.6% (1)	[234]	-	-
Populations								
Adults >=18	-	-	-	-	12.2-75.6% (1)	[234]	-	-
Children and/or adolescents	-	-	-	-	0.0-40.0% (1)	[199]	0-100% (1)	[235]
Adults & Children and/or adolescents	-	-	-	-	0.0-75.6% (2)	[199,234]		
Influenza type A								
H1N1	-	-	-	-	-	-	-	-
H2N3	-	-	-	-	-	-	-	-
Type A not specified	-	-	0% (1)	[24]	-	-	2% (1)	[235]
Influenza type B								
B/Yamagata	-	-	-	-	-	-	-	-
B/Victoria	-	-	-	-	-	-	-	-
B/Yamagata & B/Victoria	-	-	-	-	-	-	-	-
Type B not specified	-	-	0% (1)	[24]	-	-	1% (1)	[235]
Other stratification factors	-	-	-	-	Enrollment site (1)	[199]	Clinical manifestation (1)	[235]
**Long-term sequelae**								
No studies reported data on long-term sequelae.								

* One study reported number of cases without reporting proportions [236].

Disease burden of influenza-like illness
Incidence rate

Table S29. Summary of range of incidence rate for influenza-like illness cases.

Range (min-max), (number of studies)	Asia		Sub-Saharan Africa		Latin America		Middle East & North Africa	
	Range	Citation	Range	Citation	Range	Citation	Range	Citation
**All studies**	4980-11,690 per 100,000 persons) (3)	[29,37,103]	90-1030 per 100,000 persons) (1)	[60]	-	-	-	-
**Gender**								
Female	-	-	-	-	-	-	-	-
Male	-	-	-	-	-	-	-	-
**By year**								
2018	7190 – 11690 per 100,000 persons (2)	[37,103]	260 per 100,000 persons (1)	[60]	-	-	-	-
2019	4980 per 100,000 persons (1)	[103]	370 per 100,000 persons (1)	[60]	-	-	-	-
2020	-	-	-	-	-	-	-	-
2021	-	-	-	-	-	-	-	-
2022	-	-	-	-	-	-	-	-
**Populations**								
Adults >=18	6100 per 100,000 persons (1)	[29]	-	-	-	-	-	-
Children and/or adolescents	-	-	360-1030 per 100,000 persons (1)	[60]	-	-	-	-
Adults & Children and/or adolescents	4980-11,690 per 100,000 persons (2)	[37,103]	90-1030 per 100,000 persons) (1)	[60]	-	-	-	-
**Other stratification factors**	Study site	[29]	-	-	-	-	-	-

Disease burden of influenza-like illness
Proportion of cases

Table S30. Summary of proportion of influenza-like illness cases in the study population.

Range (min-max), (number of studies)	Asia		Sub-Saharan Africa		Latin America		Middle East & North Africa	
	Range	Citation	Range	Citation	Range	Citation	Range	Citation
**All studies***	0.3%-91.7% (9)	[29,42,102,237-242]	0.03%-100% (12)	[52,56,60,62,63,242-248]	0.0%-62.0% (2)	[75,242]	0.0%-78.0% (5)	[87,242,249-251]
**Gender**								
Female	6.1%-40% (2)	[29,241]	1.6%-63.7% (2)	[246,247]	-	-	75.0% (1)	[251]
Male	9.5%-60% (2)	[29,241]	5.6%-36.2% (2)	[246,247]	-	-	24.4% (1)	[251]
**By year**								
2018	-	-	0.9%-19.7% (2)	[60,63]	-	-	-	-
2019	-	-	1.3%-20% (2)	[60,63]	-	-	-	-
2020	-	-	-	-	-	-	1.8% (1)	[251]
2021	-	-	-	-	-	-	-	-
2022	-	-	-	-	-	-	-	-
**Populations**								
Adults >=18	4.2%-60.3% (4)	[29,237-239]	0.4%-78.4% (5)	[60,244,246-248]	54.8% (1)	[75]	8.1%-16.3% (1)	[251]
Children and/or adolescents	-	-	1.0%-48.3% (5)	[60,243,244,247,248]	-	-	6.1%-16.3% (1)	[251]
Adults & Children and/or adolescents	0.3%-91.7% (5)	[42,102,240-242]	0.03%-100% (11)	[52,56,60,62,63,242,244-248]	0.0%-62.0% (1)	[242]	0.0%-78.0% (4)	[87,242,250,251]
**Other stratification factors**	Occupation, outbreak phase, symptom, Career length, work location, working hours, protective equipment use, department, vaccination status, study site, comorbidi-ties, region (8)	[29,42,102,237-241]	Symptom, Diagnostic methods	[62,245,246]	-	-	Region, Governorate	[251]

* One study reported number of cases without reporting proportions [252].

Table S31. Summary of proportion of hospitalization associated with influenza-like illness in the study population.

Range (min-max), (number of studies)	Asia		Sub-Saharan Africa		Latin America		Middle East & North Africa	
	Range	Citation	Range	Citation	Range	Citation	Range	Citation
**All studies***	3.72% (1)	[29]	1.7%-82.0% (5)	[53,56,126,246,247]	0-0.6% (1)	[132]	-	-
**Gender**								
Female	-	-	27% (1)	[56]	-	-	-	-
Male	-	-	73% (1)	[56]	-	-	-	-
**By year**								
2018	-	-			0.6% (1)	[132]	-	-
2019	-	-	15.6% (1)	[53]	0% (1)	[132]	-	-
2020	-	-	9% (1)	[53]	-	-	-	-
2021	-	-	-	-	-	-	-	-
2022	-	-	-	-	-	-	-	-
**Populations**								
Adults >=18	-	-	2.4%-24.7% (2)	[53,126]	0-0.6% (1)	[132]	-	-
Children and/or adolescents	-	-	1.7%-32.0% (3)	[53,56,126]	-	-	-	-
Adults & Children and/or adolescents	-	-	9.0%-82.0% (4)	[53,56,246,247]	-	-	-	-
**Other stratification factors**	-	-	Ward type, primary admission diagnosis	[56]	-	-	-	-

* One study reported number of cases without reporting proportions [253].

Table S32. Summary of proportion of death associated with influenza-like illness in the study population.

Range (min-max), (number of studies)	Asia		Sub-Saharan Africa		Latin America		Middle East & North Africa	
	Range	Citation	Range	Citation	Range	Citation	Range	Citation
**All studies**	-	-	0.0%-18.0% (3)	[53,56,247]	0.8% (1)	[132]	0.0%-22.5% (2)	[251,254]
**Gender**								
Female	-	-	-	-	-	-	-	-
Male	-	-	-	-	-	-	-	-
**By year**								
2018	-	-	-	-	-	-	-	-
2019	-	-	1.8% (1)	[53]	-	-	-	-
2020	-	-	0% (1)	[53]	-	-	-	-

2021	-	-	-	-	-	-	-	-
2022	-	-	-	-	-	-	-	-
**Populations**								
Adults >=18	-	-	-	-	0.8% (1)	[132]	22.5% (1)	[254]
Children and/or adolescents	-	-	-	-	-	-	-	-
Adults & Children and/or adolescents	-	-	0.0%-18.0% (3)	[53,56,247]	-	-	0.0% (1)	[251]
**Other stratification factors**	-	-	-	-	-	-	-	-

Table S33. Summary of proportion of outpatient visits associated with influenza-like illness in the study population.

**Range (min-max), (number of studies)**	**Asia**		**Sub-Saharan Africa**		**Latin America**		**Middle East & North Africa**	
	**Range**	**Citation**	**Range**	**Citation**	**Range**	**Citation**	**Range**	**Citation**
**All studies**	7.5%-13.7% (1)	[255]	0.0%-78.0% (4)	[53,55,246,256]	23.7%-33.9% (1)	[200]	43.6% (1)	[90]
**Gender**								
Female	-	-	44.4% (1)	[246]	-	-	-	-
Male	-	-	34.0% (1)	[246]	-	-	-	-
**By year**								
2018	11.1% (1)	[255]	-	-	-	-	-	-
2019	13.7% (1)	[255]	17.1% (1)	[53]	-	-	-	-
2020	7.5% (1)	[255]	10%.0 (1)	[53]	-	-	-	-
2021	-	-	-	-	-	-	-	-
2022	-	-	-	-	-	-	-	-
**Populations**								
Adults >=18	-	-	3.0%-65.0% (3)	[53,55,246]	23.7%-33.9% (1)	[200]	-	-
Children and/or adolescents	-	-	8.0%-52.6% (4)	[53,55,246,256]	-	-	-	-
Adults & Children and/or adolescents	7.5%-11.1% (1)	[255]	0.0%-78.0%	[53,55,246,256]	-	-	43.6% (1)	[90]
**Other stratification factors**	-	-	Region, HIV status, Underlying illness, medical care visits, number of healthcare visits	[246]	-	-	-	-

References

1. Jainonthee, C.; Wang, Y.L.; Chen, C.W.K.; Jainontee, K. Air Pollution-Related Respiratory Diseases and Associated Environmental Factors in Chiang Mai, Thailand, in 2011-2020. *Trop Med Infect Dis* **2022**, *7*, doi:10.3390/tropicalmed7110341.
2. Danino, D.; Ben-Shimol, S.; van der Beek, B.A.; Givon-Lavi, N.; Avni, Y.S.; Greenberg, D.; Weinberger, D.M.; Dagan, R. Decline in Pneumococcal Disease in Young Children During the Coronavirus Disease 2019 (COVID-19) Pandemic in Israel Associated With Suppression of Seasonal Respiratory Viruses, Despite Persistent Pneumococcal Carriage: A Prospective Cohort Study. *Clin Infect Dis* **2022**, *75*, e1154-e1164, doi:10.1093/cid/ciab1014.
3. Hwang, S.H.; Lee, H.; Jung, M.; Kim, S.H.; Sung, H.K.; Oh, M.D.; Lee, J.Y. Incidence, Severity, and Mortality of Influenza During 2010-2020 in Korea: A Nationwide Study Based on the Population-Based National Health Insurance Service Database. *J Korean Med Sci* **2023**, *38*, e58, doi:10.3346/jkms.2023.38.e58.
4. Dawood, F.S.; Kittikraisak, W.; Patel, A.; Hunt, D.R.; Suntarattiwong, P.; Wesley, M.G.; Thompson, M.G.; Soto, G.; Mundhada, S.; Arriola, C.S. Incidence of influenza during pregnancy and association with pregnancy and perinatal outcomes in three middle-income countries: a multisite prospective longitudinal cohort study. *The Lancet Infectious Diseases* **2021**, *21*, 97-106.
5. Cohen, C.; Kleynhans, J.; Moyes, J.; McMorrow, M.L.; Treurnicht, F.K.; Hellferscee, O.; Mathunjwa, A.; von Gottberg, A.; Wolter, N.; Martinson, N.A. Asymptomatic transmission and high community burden of seasonal influenza in an urban and a rural community in South Africa, 2017–18 (PHIRST): a population cohort study. *The Lancet Global Health* **2021**, *9*, e863-e874.
6. Rios-Silva, M.; Trujillo, X.; Huerta, M.; Benites-Godinez, V.; Guzman-Esquivel, J.; Bricio-Barrios, J.A.; Mendoza-Cano, O.; Lugo-Radillo, A.; Murillo-Zamora, E. Reemerging Influenza Virus Infections during the Dominance of the Omicron SARS-CoV-2 Variant in Mexico. *Pathogens* **2022**, *11*, doi:10.3390/pathogens11101181.
7. Shokri, A.; Moradi, G.; Moradpour, F.; Mohamadi Bolbanabad, A.; Younesi, F.; Daftarifard, P.; Ebrazeh, A. Influenza incidence overlapped with COVID-19 or under COVID-19 control measures. *Immun Inflamm Dis* **2022**, *10*, e672, doi:10.1002/iid3.672.
8. Yerdessov, S.; Abbay, A.; Makhammajanov, Z.; Zhuzzhasarova, A.; Gusmanov, A.; Sakko, Y.; Zhakhina, G.; Mussina, K.; Syssoyev, D.; Alimbayev, A. Epidemiological characteristics and seasonal variation of measles, pertussis, and influenza in Kazakhstan between 2010-2020 years. Electron J Gen Med. 2023; 20 (1): em429. **2023**.
9. Byeon, K.H.; Kim, J.; Choi, B.Y.; Kim, J.Y.; Lee, N. Age-Period-Cohort Analysis of Influenza in Koreans: the National Health Insurance Research Database, 2009-2018. *J Korean Med Sci* **2020**, *35*, e121, doi:10.3346/jkms.2020.35.e121.
10. Gomaa, M.R.; Badra, R.; El Rifay, A.S.; Kandeil, A.; Kamel, M.N.; Abo Shama, N.M.; El-Shesheny, R.; Barakat, A.B.; Ali, M.A.; Kayali, G. Incidence and seroprevalence of seasonal influenza a viruses in Egypt: Results of a community-based cohort study. *Influenza Other Respir Viruses* **2022**, *16*, 749-755, doi:10.1111/irv.12974.
11. Salman, M.; Badar, N.; Ikram, A.; Nisar, N.; Farooq, U. Estimation of seasonal influenza disease burden using sentinel site data in Pakistan 2017-2019: A cross-sectional study. *Influenza Other Respir Viruses* **2023**, *17*, e13125, doi:10.1111/irv.13125.
12. Wei, W.E.; Fook-Chong, S.; Chen, W.K.; Chlebicki, M.P.; Gan, W.H. The impact of healthcare worker influenza vaccination on nosocomial influenza in a tertiary hospital: an ecological study. *BMC Health Serv Res* **2020**, *20*, 636, doi:10.1186/s12913-020-05490-1.

13. Tan, J.Y.; Conceicao, E.P.; Wee, L.E.; Sim, J.X.Y.; Venkatachalam, I. Reduction in respiratory viral infections among hospitalized older adults during the COVID-19 pandemic. *J Am Geriatr Soc* **2021**, *69*, 1745-1747, doi:10.1111/jgs.17179.
14. Huh, K.; Kim, Y.E.; Ji, W.; Kim, D.W.; Lee, E.J.; Kim, J.H.; Kang, J.M.; Jung, J. Decrease in hospital admissions for respiratory diseases during the COVID-19 pandemic: a nationwide claims study. *Thorax* **2021**, *76*, 939-941, doi:10.1136/thoraxjnl-2020-216526.
15. Hong, T.H.; Lee, H.S.; Kim, N.E.; Lee, K.J.; Kim, Y.K.; An, J.N.; Kim, J.H.; Kim, H.W.; Park, S. Recent Increases in Influenza-Related Hospitalizations, Critical Care Resource Use, and In-Hospital Mortality: A 10-Year Population-Based Study in South Korea. *J Clin Med* **2022**, *11*, doi:10.3390/jcm11164911.
16. Olivares Barraza, M.F.; Fasce, R.A.; Nogareda, F.; Marcenac, P.; Vergara Mallegas, N.; Bustos Alister, P.; Loayza, S.; Chard, A.N.; Arriola, C.S.; Couto, P.; et al. Influenza Incidence and Vaccine Effectiveness During the Southern Hemisphere Influenza Season - Chile, 2022. *MMWR Morb Mortal Wkly Rep* **2022**, *71*, 1353-1358, doi:10.15585/mmwr.mm7143a1.
17. Bianculli, P.M.; Bellier, L.; Mangado, I.O.; Perez, C.G.; Mieres, G.; Lazarov, L.; Petitjean, A.; Dibarboure, H.; Lopez, J.G. Switching from trivalent to quadrivalent inactivated influenza vaccines in Uruguay: a cost-effectiveness analysis. *Hum Vaccin Immunother* **2022**, *18*, 2050653, doi:10.1080/21645515.2022.2050653.
18. Bellier, L.; Petitjean, A.; Sarazu, T.; Tresierra, J.; Lopez, J.G. Cost-effectiveness analysis of switching from a trivalent to a quadrivalent inactivated influenza vaccine in the Peruvian immunisation programme. *Vaccine* **2021**, *39*, 4144-4152, doi:10.1016/j.vaccine.2021.05.084.
19. Farah, Z.; El Naja, H.A.; Tempia, S.; Saleh, N.; Abubakar, A.; Maison, P.; Ghosn, N. Estimation of the influenza-associated respiratory hospitalization burden using sentinel surveillance data, Lebanon, 2015-2020. *Influenza Other Respir Viruses* **2023**, *17*, e13138, doi:10.1111/irv.13138.
20. Kim, J.H.; Kim, H.Y.; Lee, M.; Ahn, J.G.; Baek, J.Y.; Kim, M.Y.; Huh, K.; Jung, J.; Kang, J.M. Respiratory Syncytial Virus Outbreak Without Influenza in the Second Year of the Coronavirus Disease 2019 Pandemic: A National Sentinel Surveillance in Korea, 2021-2022 Season. *J Korean Med Sci* **2022**, *37*, e258, doi:10.3346/jkms.2022.37.e258.
21. Pathak, S.; Jolly, M.K.; Nandi, D. Countries with high deaths due to flu and tuberculosis demonstrate lower COVID-19 mortality: roles of vaccinations. *Hum Vaccin Immunother* **2021**, *17*, 2851-2862, doi:10.1080/21645515.2021.1908058.
22. Arellanos-Soto, D.; Padilla-Rivas, G.; Ramos-Jimenez, J.; Galan-Huerta, K.; Lozano-Sepulveda, S.; Martinez-Acuna, N.; Trevino-Garza, C.; Montes-de-Oca-Luna, R.; de-la, O.C.M.; Rivas-Estilla, A.M. Decline in influenza cases in Mexico after the implementation of public health measures for COVID-19. *Sci Rep* **2021**, *11*, 10730, doi:10.1038/s41598-021-90329-w.
23. Zou, J. Research and prediction of the number of cases of influenza and its complications[Chinese]. **2021**.
24. Meiring, S.; Tempia, S.; Dominic, E.M.; de Gouveia, L.; McAnerney, J.; von Gottberg, A.; Cohen, C. Excess Invasive Meningococcal Disease Associated With Seasonal Influenza, South Africa, 2003-2018. *Clin Infect Dis* **2022**, *74*, 1729-1735, doi:10.1093/cid/ciab702.
25. Yang, J.R.; Kuo, C.Y.; Huang, H.Y.; Hsu, S.Z.; Wu, F.T.; Wu, F.T.; Li, C.H.; Liu, M.T. Seasonal dynamics of influenza viruses and age distribution of infected individuals across nine seasons covering 2009-2018 in Taiwan. *J Formos Med Assoc* **2020**, *119*, 850-860, doi:10.1016/j.jfma.2019.08.030.
26. Kim, M.; Yune, S.; Chang, S.; Jung, Y.; Sa, S.O.; Han, H.W. The Fever Coach Mobile App for Participatory Influenza Surveillance in Children: Usability Study. *JMIR Mhealth Uhealth* **2019**, *7*, e14276, doi:10.2196/14276.

27. Kim, J.H.; Roh, Y.H.; Ahn, J.G.; Kim, M.Y.; Huh, K.; Jung, J.; Kang, J.M. Respiratory syncytial virus and influenza epidemics disappearance in Korea during the 2020-2021 season of COVID-19. *Int J Infect Dis* **2021**, *110*, 29-35, doi:10.1016/j.ijid.2021.07.005.
28. Sun, Y.; Zhang, T.; Zhao, X.; Qian, J.; Jiang, M.; Jia, M.; Xu, Y.; Yang, W.; Feng, L. High activity levels of avian influenza upwards 2018-2022: A global epidemiological overview of fowl and human infections. *One Health* **2023**, *16*, 100511, doi:10.1016/j.onehlt.2023.100511.
29. Kittikraisak, W.; Wongrapee, T.; Punjasamanvong, S.; Piyaraj, P.; Vachiraphan, A.; Yoocharoen, P.; Klungthong, C.; Jones, A.R.; Tanathitikorn, C.; Mott, J.A.; et al. Influenza-Like Symptom Incidence, Illness-Associated Expenses, and Economic Impact Among Healthcare Personnel in Thailand: A Prospective Observational Cohort Study (2020-2021). *Ann Work Expo Health* **2023**, *67*, 330-344, doi:10.1093/annweh/wxac089.
30. Chiu, Y.T.; Tien, N.; Lin, H.C.; Wei, H.M.; Lai, H.C.; Chen, J.A.; Low, Y.Y.; Lin, H.H.; Hsu, Y.L.; Hwang, K.P. Detection of respiratory pathogens by application of multiplex PCR panel during early period of COVID-19 pandemic in a tertiary hospital in Central Taiwan. *J Microbiol Immunol Infect* **2022**, *55*, 1144-1150, doi:10.1016/j.jmii.2021.09.011.
31. Pham-Thanh, L.; Nhu, T.V.; Nguyen, T.V.; Tran, K.V.; Nguyen, K.C.; Nguyen, H.T.; Ngo Thi, H.; Padungtod, P. Zoonotic pathogens and diseases detected in Vietnam, 2020-2021. *One Health* **2022**, *14*, 100398, doi:10.1016/j.onehlt.2022.100398.
32. Jayaram, A.; Jagadesh, A.; Kumar, A.M.V.; Davtyan, H.; Thekkur, P.; Vilas, V.; Mandal, S.K.; Sudandiradas, R.; Babu, N.; Varamballi, P.; et al. Trends in Influenza Infections in Three States of India from 2015-2021: Has There Been a Change during COVID-19 Pandemic? *Trop Med Infect Dis* **2022**, *7*, doi:10.3390/tropicalmed7060110.
33. Yorsaeng, R.; Suntronwong, N.; Thongpan, I.; Chuchaona, W.; Lestari, F.B.; Pasittungkul, S.; Puenpa, J.; Atsawawaranunt, K.; Sharma, C.; Sudhinaraset, N.; et al. The impact of COVID-19 and control measures on public health in Thailand, 2020. *PeerJ* **2022**, *10*, e12960, doi:10.7717/peerj.12960.
34. Aggarwal, N.; Potdar, V.; Vijay, N.; Mukhopadhyay, L.; Borkakoty, B.; Manjusree, S.; Choudhary, M.L.; Chowdhury, D.; Verma, R.; Bhardwaj, S.D.; et al. SARS-CoV-2 and Influenza Virus Co-Infection Cases Identified through ILI/SARI Sentinel Surveillance: A Pan-India Report. *Viruses* **2022**, *14*, doi:10.3390/v14030627.
35. Lee, H.; Sung, H.K.; Lee, D.; Choi, Y.; Lee, J.Y.; Lee, J.Y.; Oh, M.D. Comparison of Complications after Coronavirus Disease and Seasonal Influenza, South Korea. *Emerg Infect Dis* **2022**, *28*, 347-353, doi:10.3201/eid2802.211848.
36. Chen, A.P.; Chuang, C.; Huang, Y.C.; Wu, P.F.; Huang, S.F.; Cheng, N.C.; Lin, Y.T.; Chen, S.J.; Huang, L.J.; Lee, C.L.; et al. The epidemiology and etiologies of respiratory tract infection in Northern Taiwan during the early phase of coronavirus disease 2019 (COVID-19) outbreak. *J Microbiol Immunol Infect* **2021**, *54*, 801-807, doi:10.1016/j.jmii.2021.05.006.
37. Darmaa, O.; Burmaa, A.; Gantsooj, B.; Darmaa, B.; Nymadawa, P.; Sullivan, S.G.; Fielding, J.E. Influenza epidemiology and burden of disease in Mongolia, 2013-2014 to 2017-2018. *Western Pac Surveill Response J* **2021**, *12*, 28-37, doi:10.5365/wpsar.2020.11.4.003.
38. Ravikanth, R. Diagnostic accuracy and false-positive rate of chest CT as compared to RT-PCR in coronavirus disease 2019 (COVID-19) pneumonia: A prospective cohort of 612 cases from India and review of literature. *Indian J Radiol Imaging* **2021**, *31*, S161-S169, doi:10.4103/ijri.IJRI_377_20.
39. Baral, S.D.; Rucinski, K.B.; Twahirwa Rwema, J.O.; Rao, A.; Prata Menezes, N.; Diouf, D.; Kamarulzaman, A.; Phaswana-Mafuya, N.; Mishra, S. The Relationship Between the Global Burden of Influenza From 2017 to 2019 and COVID-19: Descriptive Epidemiological Assessment. *JMIR Public Health Surveill* **2021**, *7*, e24696, doi:10.2196/24696.

40. Suntronwong, N.; Vichaiwattana, P.; Klinfueng, S.; Korkong, S.; Thongmee, T.; Vongpunsawad, S.; Poovorawan, Y. Climate factors influence seasonal influenza activity in Bangkok, Thailand. *PLoS One* **2020**, *15*, e0239729, doi:10.1371/journal.pone.0239729.
41. Siegers, J.Y.; Dhanasekaran, V.; Xie, R.; Deng, Y.M.; Patel, S.; Ieng, V.; Moselen, J.; Peck, H.; Aziz, A.; Sarr, B.; et al. Genetic and Antigenic Characterization of an Influenza A(H3N2) Outbreak in Cambodia and the Greater Mekong Subregion during the COVID-19 Pandemic, 2020. *J Virol* **2021**, *95*, e0126721, doi:10.1128/JVI.01267-21.
42. Sovann, L.Y.; Sar, B.; Kab, V.; Yann, S.; Kinzer, M.; Raftery, P.; Albalak, R.; Patel, S.; Hay, P.L.; Seng, H.; et al. An influenza A (H3N2) virus outbreak in the Kingdom of Cambodia during the COVID-19 pandemic of 2020. *Int J Infect Dis* **2021**, *103*, 352-357, doi:10.1016/j.ijid.2020.11.178.
43. Valtonen, M.; Waris, M.; Vuorinen, T.; Eerola, E.; Hakanen, A.J.; Mjosund, K.; Gronroos, W.; Heinonen, O.J.; Ruuskanen, O. Common cold in Team Finland during 2018 Winter Olympic Games (PyeongChang): epidemiology, diagnosis including molecular point-of-care testing (POCT) and treatment. *Br J Sports Med* **2019**, *53*, 1093-1098, doi:10.1136/bjsports-2018-100487.
44. Suntronwong, N.; Thongpan, I.; Chuchaona, W.; Budi Lestari, F.; Vichaiwattana, P.; Yorsaeng, R.; Pasittungkul, S.; Kitphati, R.; Vongpunsawad, S.; Poovorawan, Y. Impact of COVID-19 public health interventions on influenza incidence in Thailand. *Pathog Glob Health* **2020**, *114*, 225-227, doi:10.1080/20477724.2020.1777803.
45. Soo, R.J.J.; Chiew, C.J.; Ma, S.; Pung, R.; Lee, V. Decreased Influenza Incidence under COVID-19 Control Measures, Singapore. *Emerg Infect Dis* **2020**, *26*, 1933-1935, doi:10.3201/eid2608.201229.
46. Sharma, S.; Patel, P.; Kulkarni, S.V.; Deoshatwar, A.; Yadav, R.; Tanwar, S.; Dolla, J.R.; Jain, S.K.; Singh, S.K.; Dikid, T. An outbreak of acute neurological illness associated with drinking water source following a cyclone in Eluru, West Godavari district, Andhra Pradesh, India, December 2020. *Clinical Epidemiology and Global Health* **2023**, *20*, 101261.
47. Berry, I.; Rahman, M.; Flora, M.S.; Shirin, T.; Alamgir, A.; Khan, M.H.; Anwar, R.; Lisa, M.; Chowdhury, F.; Islam, M.A. Seasonality of influenza and coseasonality with avian influenza in Bangladesh, 2010–19: a retrospective, time-series analysis. *The Lancet Global Health* **2022**, *10*, e1150-e1158.
48. World Health Organization. Global Influenza Programme: Influenza surveillance outputs. **2023**.
49. Lingani, M.; Cisse, A.; Tialla, D.; Ilboudo, A.K.; Savadogo, M.; Sawadogo, C.; Gampini, S.; Tarnagda, G.; Tao, M.; Diagbouga, S.; et al. Coinfections with SARS-CoV-2 variants and influenza virus during the 2019 Coronavirus disease pandemic in Burkina Faso: A surveillance study. *Health Sci Rep* **2023**, *6*, e1041, doi:10.1002/hsr2.1041.
50. Moumbeket Yifomnjou, M.H.; Monamele, G.C.; Njankouo-Ripa, M.; Fatawou Modiyinji, A.; Ngoupo, P.A.; Boyomo, O.; Njouom, R. Viral co-infection with human respiratory syncytial virus in suspected acute and severe respiratory tract infections during COVID-19 pandemic in Yaounde, Cameroon, 2020-2021. *Influenza Other Respir Viruses* **2023**, *17*, e13131, doi:10.1111/irv.13131.
51. Shedura, V.J.; Hussein, A.K.; Nyanga, S.K.; Kamori, D.; McHau, G.J. Evaluation of the influenza-like illness sentinel surveillance system: A national perspective in Tanzania from January to December 2019. *PLoS One* **2023**, *18*, e0283043, doi:10.1371/journal.pone.0283043.
52. Goumballa, N.; Sambou, M.; Samba, D.F.; Bassene, H.; Bedotto, M.; Aidara, A.; Dieng, M.; Hoang, V.T.; Parola, P.; Sokhna, C.; et al. PCR investigation of infections in patients consulting at a healthcare centre over a four-year period during the Grand Magal of Touba. *Travel Med Infect Dis* **2023**, *52*, 102515, doi:10.1016/j.tmaid.2022.102515.

53. Loevinsohn, G.; Hamahuwa, M.; Hardick, J.; Sinywimaanzi, P.; Fenstermacher, K.Z.J.; Munachoonga, P.; Weynand, A.; Monze, M.; Manabe, Y.C.; Gaydos, C.A.; et al. Respiratory viruses in rural Zambia before and during the COVID-19 pandemic. *Trop Med Int Health* **2022**, *27*, 647-654, doi:10.1111/tmi.13781.
54. Toure, C.T.; Fall, A.; Andriamandimby, S.F.; Jallow, M.M.; Goudiaby, D.; Kiori, D.; Sy, S.; Diaw, Y.; Ndiaye, K.N.; Mbaye, F.; et al. Epidemiology and Molecular Analyses of Influenza B Viruses in Senegal from 2010 to 2019. *Viruses* **2022**, *14*, doi:10.3390/v14051063.
55. Loevinsohn, G.; Hamahuwa, M.; Sinywimaanzi, P.; Fenstermacher, K.Z.J.; Shaw-Saliba, K.; Pekosz, A.; Monze, M.; Rothman, R.E.; Simulundu, E.; Thuma, P.E.; et al. Facility-based surveillance for influenza and respiratory syncytial virus in rural Zambia. *BMC Infect Dis* **2021**, *21*, 986, doi:10.1186/s12879-021-06677-5.
56. Loevinsohn, G.; Hardick, J.; Mehoke, T.; Sinywimaanzi, P.; Hamahuwa, M.; Fenstermacher, K.Z.J.; Shaw-Saliba, K.; Thielen, P.; Evans, J.; Bowden, K.; et al. Nosocomial Respiratory Infections in a Rural Zambian Hospital. *Am J Trop Med Hyg* **2021**, *105*, 818-821, doi:10.4269/ajtmh.20-1470.
57. Cohen, C.; McMorrow, M.L.; Martinson, N.A.; Kahn, K.; Treurnicht, F.K.; Moyes, J.; Mkhencele, T.; Hellferscee, O.; Lebina, L.; Moroe, M.; et al. Cohort profile: A Prospective Household cohort study of Influenza, Respiratory syncytial virus and other respiratory pathogens community burden and Transmission dynamics in South Africa, 2016-2018. *Influenza Other Respir Viruses* **2021**, *15*, 789-803, doi:10.1111/irv.12881.
58. Wang, H.; Zhao, J.; Xie, N.; Wang, W.; Qi, R.; Hao, X.; Liu, Y.; Sevalie, S.; Niu, G.; Zhang, Y.; et al. A Prospective Study of Etiological Agents Among Febrile Patients in Sierra Leone. *Infect Dis Ther* **2021**, *10*, 1645-1664, doi:10.1007/s40121-021-00474-y.
59. Nzoumbou-Boko, R.; Yambiyo, B.M.; Ngoagouni, C.; Vickos, U.; Manirakiza, A.; Nakoune, E. Falciparum Malaria in Febrile Patients at Sentinel Sites for Influenza Surveillance in the Central African Republic from 2015 to 2018. *Interdiscip Perspect Infect Dis* **2020**, *2020*, 3938541, doi:10.1155/2020/3938541.
60. Monamele, C.G.; Messanga Essengue, L.L.; Ripa Njankouo, M.; Munshili Njifon, H.L.; Tchatchueng, J.; Tejiokem, M.C.; Njouom, R. Evaluation of a mobile health approach to improve the Early Warning System of influenza surveillance in Cameroon. *Influenza Other Respir Viruses* **2020**, *14*, 491-498, doi:10.1111/irv.12747.
61. Njouom, R.; Monamele, C.G.; Munshili Njifon, H.L.; Kenmoe, S.; Ripa Njankouo, M.; Network of influenza surveillance in, C. Circulation of influenza virus from 2009 to 2018 in Cameroon: 10 years of surveillance data. *PLoS One* **2019**, *14*, e0225793, doi:10.1371/journal.pone.0225793.
62. Kelly, M.E.; Gharpure, R.; Shivji, S.; Matonya, M.; Moshi, S.; Mwafulango, A.; Mwalongo, V.; Mghamba, J.; Simba, A.; Balajee, S.A.; et al. Etiologies of influenza-like illness and severe acute respiratory infections in Tanzania, 2017-2019. *PLOS Glob Public Health* **2023**, *3*, e0000906, doi:10.1371/journal.pgph.0000906.
63. Asante, I.A.; Fox, A.T.; Behene, E.; Awuku-Larbi, Y.; Kotey, E.N.; Nyarko, S.; Obeng, R.A.; Arjarquah, A.; Mawuli, G.; Magnusen, V.; et al. Epidemiology of influenza in Ghana, 2011 to 2019. *PLOS Glob Public Health* **2022**, *2*, e0001104, doi:10.1371/journal.pgph.0001104.
64. Loevinsohn, G.; Hardick, J.; Sinywimaanzi, P.; Fenstermacher, K.Z.J.; Shaw-Saliba, K.; Monze, M.; Gaydos, C.A.; Rothman, R.E.; Pekosz, A.; Thuma, P.E.; et al. Respiratory pathogen diversity and co-infections in rural Zambia. *Int J Infect Dis* **2021**, *102*, 291-298, doi:10.1016/j.ijid.2020.10.054.
65. Adema, I.W.; Kamau, E.; Uchi Nyiro, J.; Otieno, G.P.; Lewa, C.; Munywoki, P.K.; Nokes, D.J. Surveillance of respiratory viruses among children attending a primary school in rural coastal Kenya. *Wellcome Open Res* **2020**, *5*, 63, doi:10.12688/wellcomeopenres.15703.2.

66. Otieno, N.A.; Azziz-Baumgartner, E.; Nyawanda, B.O.; Oreri, E.; Ellington, S.; Onyango, C.; Emukule, G.O. SARS-CoV-2 Infection among Pregnant and Postpartum Women, Kenya, 2020-2021. *Emerg Infect Dis* **2021**, *27*, 2497-2499, doi:10.3201/eid2709.210849.
67. El Zowalaty, M.E.; Abdelgadir, A.; Borkenhagen, L.K.; Ducatez, M.F.; Bailey, E.S.; Gray, G.C. Influenza A viruses are likely highly prevalent in South African swine farms. *Transbound Emerg Dis* **2022**, *69*, 2373-2383, doi:10.1111/tbed.14255.
68. Asante, I.A.; Hsu, S.N.; Boatemaa, L.; Kwasah, L.; Adusei-Poku, M.; Odoom, J.K.; Awuku-Larbi, Y.; Foulkes, B.H.; Oliver-Commey, J.; Asiedu, E.K. Repurposing an integrated national influenza platform for genomic surveillance of SARS-CoV-2 in Ghana: a molecular epidemiological analysis. *The Lancet Global Health* **2023**, *11*, e1075-e1085.
69. Anjorin, A.A.A.; Nwammadu, J.E. Seroepidemiology of seasonal influenza virus among unvaccinated pregnant women in Lagos, Nigeria. *Infez Med* **2020**, *28*, 407-415.
70. Ministry of Health Kingdom of Eswatini. *First quarter performance report for 2019-20* 2020.
71. Correia, W.; Dorta-Guerra, R.; Sanches, M.; Almeida Semedo, C.d.J.B.; Valladares, B.; de Pina-Araujo, I.I.M.; Carmelo, E. Study of the etiology of acute respiratory infections in children under 5 years at the Dr. Agostinho Neto Hospital, Praia, Santiago Island, Cabo Verde. *Frontiers in Pediatrics* **2021**, *9*, 716351.
72. Keita, M.B.; Pierre, F.; Ndjomou, J.; Traoré, B.; Tohonamou, P.; Soumaré, M.; Mamadi, S.; Keita, M.A.; Bile, C.E.; Pallawo, R.B. The first epidemiological and virological influenza surveillance in the Republic of Guinea revealed the predominance of influenza A/H3N2 and B Victoria viruses. *Epidemiology & Infection* **2021**, *149*, e223.
73. Loevinsohn G; Mehoke T; Sinywimaanzi P. Influenza and RSV infection in rural Zambia **2020**.
74. Lima, T.M.; Palamim, C.V.C.; Melani, V.F.; Mendes, M.F.; Pereira, L.R.; Marson, F.A.L. COVID-19 Underreporting in Brazil among Patients with Severe Acute Respiratory Syndrome during the Pandemic: An Ecological Study. *Diagnostics (Basel)* **2022**, *12*, doi:10.3390/diagnostics12061505.
75. Owusu, D.; Dawood, F.S.; Azziz-Baumgartner, E.; Tinoco, Y.; Soto, G.; Gonzalez, O.; Cabrera, S.; Florian, R.; Llajaruna, E.; Hunt, D.R.; et al. Effectiveness of Maternal Influenza Vaccination in Peru PRIME Cohort. *Open Forum Infect Dis* **2023**, *10*, ofad033, doi:10.1093/ofid/ofad033.
76. Costa, V.G.D.; Gomes, A.J.C.; Bittar, C.; Geraldini, D.B.; Previdelli da Conceicao, P.J.; Cabral, A.S.; Carvalho, T.; Biselli, J.M.; Provazzi, P.J.S.; Campos, G.R.F.; et al. Burden of Influenza and Respiratory Syncytial Viruses in Suspected COVID-19 Patients: A Cross-Sectional and Meta-Analysis Study. *Viruses* **2023**, *15*, doi:10.3390/v15030665.
77. Zarur-Torralvo, S.; Stand-Nino, I.; Florez-Garcia, V.; Mendoza, H.; Viana-Cardenas, E. Viruses responsible for acute respiratory infections before (2016-2019) and during (2021) circulation of the SARS-CoV-2 virus in pediatric patients in a reference center at Barranquilla Colombia: A pattern analysis. *J Med Virol* **2023**, *95*, e28439, doi:10.1002/jmv.28439.
78. Costa, J.C.D.; Siqueira, M.M.; Brown, D.; Lopes, J.O.; Costa, B.C.D.; Gama, E.L.; Aguiar-Oliveira, M.L. Vaccine Mismatches, Viral Circulation, and Clinical Severity Patterns of Influenza B Victoria and Yamagata Infections in Brazil over the Decade 2010-2020: A Statistical and Phylogeny-Trait Analyses. *Viruses* **2022**, *14*, doi:10.3390/v14071477.
79. Varela, F.H.; Sartor, I.T.S.; Polese-Bonatto, M.; Azevedo, T.R.; Kern, L.B.; Fazolo, T.; de David, C.N.; Zavaglia, G.O.; Fernandes, I.R.; Krauser, J.R.M.; et al. Rhinovirus as the main co-circulating virus during the COVID-19 pandemic in children. *J Pediatr (Rio J)* **2022**, *98*, 579-586, doi:10.1016/j.jped.2022.03.003.

80. Leal, L.F.; Merckx, J.; Fell, D.B.; Kuchenbecker, R.; Miranda, A.E.; de Oliveira, W.K.; Platt, R.W.; Antunes, L.; Silveira, M.F.; Barbieri, N.B. Characteristics and outcomes of pregnant women with SARS-CoV-2 infection and other severe acute respiratory infections (SARI) in Brazil from January to November 2020. *Braz J Infect Dis* **2021**, *25*, 101620, doi:10.1016/j.bjid.2021.101620.
81. Varela, F.H.; Scotta, M.C.; Polese-Bonatto, M.; Sartor, I.T.S.; Ferreira, C.F.; Fernandes, I.R.; Zavaglia, G.O.; de Almeida, W.A.F.; Arakaki-Sanchez, D.; Pinto, L.A.; et al. Absence of detection of RSV and influenza during the COVID-19 pandemic in a Brazilian cohort: Likely role of lower transmission in the community. *J Glob Health* **2021**, *11*, 05007, doi:10.7189/jogh.11.05007.
82. Gregianini, T.S.; Varella, I.R.S.; Fisch, P.; Martins, L.G.; Veiga, A.B.G. Dual and Triple Infections With Influenza A and B Viruses: A Case-Control Study in Southern Brazil. *J Infect Dis* **2019**, *220*, 961-968, doi:10.1093/infdis/jiz221.
83. Novaes, T.E.R.; Lara, D.M.; da Silva, S.G. Severe Acute Respiratory Syndrome (SARS) in the Context of the COVID-19 Pandemic Among Indigenous Peoples of Brazil: Epidemiology and Risk Factors Associated with Death. *J Racial Ethn Health Disparities* **2023**, doi:10.1007/s40615-023-01660-z.
84. Lucas, P.C.C.; Lorenz, C.; Florez-Montero, G.L.; Palasio, R.G.S.; Portella, T.P.; Monteiro, P.C.M.; Yu, A.L.F.; Carvalhanas, T. Institutional outbreaks of influenza-like illnesses in the state of Sao Paulo: an analysis of the epidemiological profile during the COVID-19 pandemic. *Public Health* **2023**, *221*, 142-149, doi:10.1016/j.puhe.2023.06.018.
85. Taktak, A.; Smaoui, F.; Chtourou, A.; Maaloul, M.; Karray-Hakim, H.; Hammami, A.; Fki-Berrajah, L.; Gargouri, S. Significant impact of COVID-19 pandemic on the circulation of respiratory viruses in Tunisia, 2020-2021. *Clin Epidemiol Glob Health* **2023**, *21*, 101306, doi:10.1016/j.cegh.2023.101306.
86. Kandeel, A.; Fahim, M.; Deghedy, O.; Alim, W.; Roshdy, W.H.; Khalifa, M.K.; El Shesheny, R.; Kandeil, A.; Naguib, A.; Elguindy, N.; et al. Incidence, risk factors, and whole-genome sequence of SARs-CoV-2 and influenza virus among the Egyptian pilgrims returning from Umrah mass gathering in Saudi Arabia, April-May 2022. *J Infect Public Health* **2022**, *15*, 1290-1296, doi:10.1016/j.jiph.2022.10.005.
87. Fahim, M.; Roshdy, W.H.; Deghedy, O.; Kamel, R.; Naguib, A.; Showky, S.; Elguindy, N.; Abdel Fattah, M.; Afifi, S.; Mohsen, A.; et al. Epidemiology, Disease Severity and Outcome of Severe Acute Respiratory Syndrome Coronavirus 2 and Influenza Viruses Coinfection Seen at Egypt Integrated Acute Respiratory Infections Surveillance, 2020-2022. *Can J Infect Dis Med Microbiol* **2022**, *2022*, 7497500, doi:10.1155/2022/7497500.
88. Al Amad, M.; Almoayed, K. Influenza circulating viruses, positivity rate and risk factors for influenza associated severe acute respiratory infection during 2018/2019 winter season, Yemen. *BMC Infect Dis* **2022**, *22*, 111, doi:10.1186/s12879-022-07090-2.
89. Gharieb, R.; Mohamed, M.; Khalil, A.; Ali, A. Influenza A viruses in birds and humans: Prevalence, molecular characterization, zoonotic significance and risk factors' assessment in poultry farms. *Comp Immunol Microbiol Infect Dis* **2019**, *63*, 51-57, doi:10.1016/j.cimid.2019.01.001.
90. Fahim, M.; Abu ElSood, H.; AbdElGawad, B.; Deghedy, O.; Naguib, A.; Roshdy, W.H.; Showky, S.; Kamel, R.; Elguindy, N.; Abdel Fattah, M.; et al. Adapting an integrated acute respiratory infections sentinel surveillance to the COVID-19 pandemic requirements, Egypt, 2020-2022. *Public Health Pract (Oxf)* **2023**, *5*, 100358, doi:10.1016/j.puhip.2023.100358.
91. Abo Shama, N.M.; Mahmoud, S.H.; Bagato, O.; AbdElsalam, E.T.; Alkhazindar, M.; Kandeil, A.; McKenzie, P.P.; Webby, R.J.; Ali, M.A.; Kayali, G.; et al. Incidence and neutralizing antibody seroprevalence of influenza B virus in Egypt: Results of a community-based cohort study. *PLoS One* **2022**, *17*, e0269321, doi:10.1371/journal.pone.0269321.

92. Rezaee, D.; Bakhtiari, S.; Jalilian, F.A.; Doosti-Irani, A.; Asadi, F.T.; Ansari, N. Coinfection with severe acute respiratory syndrome coronavirus 2 (SARS-CoV-2) and influenza virus during the COVID-19 pandemic. *Arch Virol* **2023**, *168*, 53, doi:10.1007/s00705-022-05628-y.
93. Abou El Naja, H.; Tempia, S.; Barakat, A.; Elkholy, A.; Aman, A.; Khan, W.; Abubakar, A. Influenza activity in the Eastern Mediterranean Region (EMR) in 2020–2021 amidst the COVID-19 pandemic. *BMJ Global Health* **2022**, *7*, e008506.
94. Khoshakhlagh, M.; Vojdani, A.; Amali, A.; Abolbashari, S.; Gholoobi, A.; Meshkat, Z. Evaluation of Viral Respiratory Pathogens Among Patients Initially Tested Negative for SARS-CoV-2. *Jundishapur Journal of Microbiology* **2023**, *16*.
95. Low, Y.L.; Wong, S.Y.; Lee, H.E.K.; Muhammed, M.H. Epidemiology of Respiratory Viruses in Acute Respiratory Illnesses in Malaysia: Patterns, Seasonality and Age Distribution. *medRxiv* **2022**, 2022.2003. 2003.22271672.
96. Duman, N.; Sarınoğlu, R.C.; Kuzan, B.N.; Güncü, M.M.; Aslan, B.; Cimşit, N.Ç.; Karahasan, A. Evaluation of BioFire Filmarray panel for respiratory pathogens: a demographic and clinical analysis in Istanbul, Turkey. *Infectio* **2023**, 86-93.
97. Klivleyeva, N.; Lukmanova, G.; Glebova, T.; Shamenova, M.; Ongarbayeva, N.; Saktaganov, N.; Baimukhametova, A.; Baiseiit, S.; Ismagulova, D.; Kassymova, G.; et al. Spread of Pathogens Causing Respiratory Viral Diseases Before and During CoVID-19 Pandemic in Kazakhstan. *Indian J Microbiol* **2023**, *63*, 129-138, doi:10.1007/s12088-023-01064-x.
98. Muruganandam, N.; Roy, A.; Sivanandan, N.; Vins, A.; Beniwal, N.; Kaur, H.; Potdar, V.; Parvez, R. Respiratory viruses among ethnic Nicobarese during COVID-19 pandemic. *BMC Infect Dis* **2022**, *22*, 463, doi:10.1186/s12879-022-07435-x.
99. Lima, A.K.S.; Banho, C.A.; Sacchetto, L.; de Carvalho Marques, B.; Dos Santos, M.G.; Ribeiro, M.R.; Gandolfi, F.A.; Sakomura, T.P.; Estofolete, C.F.; Nogueira, M.L. Seasonal respiratory virus trends in pediatric patients during the COVID-19 pandemic in Brazil. *Braz J Microbiol* **2023**, *54*, 1827-1834, doi:10.1007/s42770-023-01087-y.
100. Bhardwaj, S.; Choudhary, M.L.; Jadhav, S.; Vipat, V.; Ghuge, R.; Salvi, S.; Kulkarni, R.; Kinikar, A.; Padbidri, V.; Bafna, S.; et al. A retrospective analysis of respiratory virus transmission before and during the COVID-19 pandemic in Pune the western region of India. *Front Public Health* **2022**, *10*, 936634, doi:10.3389/fpubh.2022.936634.
101. Ndeh, N.T.; Tesfaldet, Y.T.; Budnard, J.; Chuaicharoen, P. The secondary outcome of public health measures amidst the COVID-19 pandemic in the spread of other respiratory infectious diseases in Thailand. *Travel Med Infect Dis* **2022**, *48*, 102348, doi:10.1016/j.tmaid.2022.102348.
102. Sona, S.; Sharma, A.; Chamuah, K.; Henbi, L.N.; Rajbongshi, G. Influenza virus and its subtypes circulating during 2018-2019: A hospital-based study from Assam. *Indian J Med Microbiol* **2022**, *40*, 525-530, doi:10.1016/j.ijmmb.2022.08.001.
103. Lee, H.; Lee, H.; Song, K.H.; Kim, E.S.; Park, J.S.; Jung, J.; Ahn, S.; Jeong, E.K.; Park, H.; Kim, H.B. Impact of Public Health Interventions on Seasonal Influenza Activity During the COVID-19 Outbreak in Korea. *Clin Infect Dis* **2021**, *73*, e132-e140, doi:10.1093/cid/ciaa672.
104. Sahak, M.N.; Arifi, F.; Hammond, A.A.; Laurenson-Schafer, H.J.; Saeedzai, S.A.; Safi, H.; Abubakar, A.; Elkholy, A.; Rasooly, M.H.; Ikram, A.N. The 2018-19 influenza season in Afghanistan: Epidemiology and Virology. **2020**.
105. Pang, Y.K.; Ismail, A.I.; Chan, Y.F.; Cheong, A.; Chong, Y.M.; Doshi, P.; Lau, J.Z.H.; Khor, J.; Wang, L.P.L.; Leong, C.L.; et al. Influenza in Malaysian adult patients hospitalized with community-acquired pneumonia, acute exacerbation of chronic obstructive pulmonary disease or asthma: a multicenter, active surveillance study. *BMC Infect Dis* **2021**, *21*, 644, doi:10.1186/s12879-021-06360-9.

106. Guo, M.M.; Yang, K.D.; Liu, S.F.; Kuo, H.C. Number of Kawasaki Disease Admissions Is Associated with Number of Domestic COVID-19 and Severe Enterovirus Case Numbers in Taiwan. *Children (Basel)* **2022**, *9*, doi:10.3390/children9020149.
107. Neighbors, C.E.; Myers, E.R.; Weerasinghe, N.P.; Wijayaratne, G.B.; Bodinayake, C.K.; Nagahawatte, A.; Tillekeratne, L.G.; Woods, C.W. Influenza Vaccination Implementation in Sri Lanka: A Cost-Effectiveness Analysis. *Vaccines (Basel)* **2023**, *11*, doi:10.3390/vaccines11050932.
108. Lim, K.J.; John, J.L.; Rahim, S.; Avoi, R.; Hassan, M.R.; Jeffree, M.S.; Ibrahim, M.Y.; Ahmed, K. A 1-year cross-sectional study on the predominance of influenza among hospitalized children in a tropical area, Kota Kinabalu, Sabah. *J Physiol Anthropol* **2022**, *41*, 11, doi:10.1186/s40101-022-00285-1.
109. Tan, M.P.; Leong, C.L.; Pang, Y.K.; Razali, R.M.; Ismail, A.I.; Sam, I.C.; Abdul Rani, R.; Chong, J.; Mohd Zim, M.A.; Musa, A.N.; et al. Dearth of influenza among older adults admitted with respiratory symptoms in Malaysia during the coronavirus disease 2019 pandemic in 2021. *Front Med (Lausanne)* **2022**, *9*, 977614, doi:10.3389/fmed.2022.977614.
110. Akhtar, Z.; Islam, M.A.; Aleem, M.A.; Mah, E.M.S.; Ahmmed, M.K.; Ghosh, P.K.; Rahman, M.; Rahman, M.Z.; Sumiya, M.K.; Rahman, M.M.; et al. SARS-CoV-2 and influenza virus coinfection among patients with severe acute respiratory infection during the first wave of COVID-19 pandemic in Bangladesh: a hospital-based descriptive study. *BMJ Open* **2021**, *11*, e053768, doi:10.1136/bmjopen-2021-053768.
111. Chong, Y.M.; Chan, Y.F.; Jamaluddin, M.F.H.; Hasan, M.S.; Pang, Y.K.; Ponnampalavanar, S.; Syed Omar, S.F.; Voon, K.G.L.; Sam, I.C. Detection of respiratory viruses in adults with suspected COVID-19 in Kuala Lumpur, Malaysia. *J Clin Virol* **2021**, *145*, 105000, doi:10.1016/j.jcv.2021.105000.
112. Pannu, A.K.; Kumar, M.; Singh, P.; Shaji, A.; Ghosh, A.; Behera, A.; Sharda, S.C.; Bhatia, M.; Singla, N.; Dhibar, D.P.; et al. Severe Acute Respiratory Infection Surveillance during the Initial Phase of the COVID-19 Outbreak in North India: A Comparison of COVID-19 to Other SARI Causes. *Indian J Crit Care Med* **2021**, *25*, 761-767, doi:10.5005/jp-journals-10071-23882.
113. Akhtar, Z.; Chowdhury, F.; Aleem, M.A.; Ghosh, P.K.; Rahman, M.; Rahman, M.; Hossain, M.E.; Sumiya, M.K.; Islam, A.; Uddin, M.J.; et al. Undiagnosed SARS-CoV-2 infection and outcome in patients with acute MI and no COVID-19 symptoms. *Open Heart* **2021**, *8*, doi:10.1136/openhrt-2021-001617.
114. Pham, T.D.; Hoang, V.T.; Dao, T.L.; Tran, X.D.; Phi, D.L.; To, M.M.; Dang, V.N.; Dang, V.K.; Dao, T.T.; Nguyen, N.T.; et al. Morbidity and Mortality Patterns in Children Admitted to Hospital in Thai Binh, Vietnam: A Five-year Descriptive Study with a Focus on Infectious Diseases. *J Epidemiol Glob Health* **2021**, *11*, 69-75, doi:10.2991/jegh.k.200723.001.
115. Acharya, U.; Acharya, S.P. Clinical Outcome of Patients Hospitalized in a Tertiary Care Hospital of Nepal with Confirmed Influenza A/pdm 09(H1N1) in 2018/2019. *J Nepal Health Res Counc* **2020**, *18*, 196-200, doi:10.33314/jnhrc.v18i2.2380.
116. Vanderburg, S.; Wijayaratne, G.; Danthanarayana, N.; Jayamaha, J.; Piyasiri, B.; Halloluwa, C.; Sheng, T.; Amarasena, S.; Kurukulasooriya, R.; Nicholson, B.P.; et al. Outbreak of severe acute respiratory infection in Southern Province, Sri Lanka in 2018: a cross-sectional study. *BMJ Open* **2020**, *10*, e040612, doi:10.1136/bmjopen-2020-040612.
117. Hsih, W.H.; Cheng, M.Y.; Ho, M.W.; Chou, C.H.; Lin, P.C.; Chi, C.Y.; Liao, W.C.; Chen, C.Y.; Leong, L.Y.; Tien, N.; et al. Featuring COVID-19 cases via screening symptomatic patients with epidemiologic link during flu season in a medical center of central Taiwan. *J Microbiol Immunol Infect* **2020**, *53*, 459-466, doi:10.1016/j.jmii.2020.03.008.
118. Sathyamurthy, P.; Dhandapani, N.S. Evaluation of Pregnancy, Younger Age, and Old Age as Independent Risk Factors for Poor Hospitalization Outcomes in Influenza A (H1N1)pdm09 Virus a Decade After the Pandemic. *Cureus* **2020**, *12*, e11762, doi:10.7759/cureus.11762.

119. Tai, C.C.; Tsai, C.H.; Huang, Y.H.; Lee, C.L.; Chen, H.P.; Chan, Y.J. Detection of respiratory viruses in adults with respiratory tract infection using a multiplex PCR assay at a tertiary center. *J Microbiol Immunol Infect* **2021**, *54*, 858-864, doi:10.1016/j.jmii.2020.07.020.
120. Tambunan, A.L.; Ritonga, D.H.; Al Anas, M. Comparative Demographics of Influenza A by Genetic Subtypes through Surveillance Approach for Severe Acute Respiratory Infection in Children: A Hospital-Based Study from 2019 to 2020. *International Medical Journal* **2022**, *29*.
121. Thakur, C.; Singh, D.; Sharma, A.; Kumar, V.; Kanga, A. A Concurrent Comparison of the Epidemiology and Clinical Presentation of Patients Hospitalized with Pandemic 2009 (H1N1) Influenza and Seasonal Influenza-A in Sub-himalayan Region of Himachal Pradesh. *The Journal of the Association of Physicians of India* **2019**, *67*, 70-74.
122. Andrew, M.K.; Pott, H.; Staadegaard, L.; Paget, J.; Chaves, S.S.; Ortiz, J.R.; McCauley, J.; Bresee, J.; Nunes, M.C.; Baumeister, E. Age differences in comorbidities, presenting symptoms, and outcomes of influenza illness requiring hospitalization: A worldwide perspective from the global influenza hospital surveillance network. In Proceedings of the Open Forum Infectious Diseases, 2023; p. ofad244.
123. Sivasankari, D.; Kathirvelan, J.J.; Jacob, E.S. Incidence of SARS COV-2 & Influenza A in SARI Patients Attending Tertiary Care Hospital, Thanjavur. *International Journal of Current Microbiology and Applied Sciences* **2021**, *10*, 688-696.
124. Quéromès, G.; Frobert, E.; Burtseva, E.; Drăgănescu, A.; Koul, P.A.; Komissarov, A.; Laguna-Torres, V.A.; Leblanc, J.; López-Labrador, F.-X.; Medić, S. Clinical and phylogenetic influenza dynamics for the 2019-20 season in the global influenza hospital surveillance network (GIHSN)–Pilot study. *Journal of Clinical Virology* **2022**, *152*, 105184.
125. Jarju, S.; Senghore, E.; Brotherton, H.; Affleck, L.; Saidykhan, A.; Jallow, S.; Krubally, E.; Sinjanka, E.; Ndene, M.N.; Bajo, F.; et al. Circulation of respiratory viruses during the COVID-19 pandemic in The Gambia. *Gates Open Res* **2022**, *6*, 148, doi:10.12688/gatesopenres.14155.3.
126. Tempia, S.; Walaza, S.; Bhiman, J.N.; McMorrow, M.L.; Moyes, J.; Mkhencele, T.; Meiring, S.; Quan, V.; Bishop, K.; McAnerney, J.M.; et al. Decline of influenza and respiratory syncytial virus detection in facility-based surveillance during the COVID-19 pandemic, South Africa, January to October 2020. *Euro Surveill* **2021**, *26*, doi:10.2807/1560-7917.ES.2021.26.29.2001600.
127. Wadilo, F.; Feleke, A.; Gebre, M.; Mihret, W.; Seyoum, T.; Melaku, K.; Howe, R.; Mulu, A.; Mihret, A. Viral etiologies of lower respiratory tract infections in children < 5 years of age in Addis Ababa, Ethiopia: a prospective case-control study. *Virol J* **2023**, *20*, 163, doi:10.1186/s12985-023-02131-x.
128. Izu, A.; Nunes, M.C.; Solomon, F.; Baillie, V.; Serafin, N.; Verwey, C.; Moore, D.P.; Laubscher, M.; Ncube, M.; Olwagen, C. All-cause and pathogen-specific lower respiratory tract infection hospital admissions in children younger than 5 years during the COVID-19 pandemic (2020–22) compared with the pre-pandemic period (2015–19) in South Africa: an observational study. *The Lancet Infectious Diseases* **2023**, *23*, 1031-1041.
129. Loevinsohn G; Mehoke T; P, S. Influenza and RSV infection in rural Zambia. **2020**.
130. Lina, B.; Georges, A.; Burtseva, E.; Nunes, M.C.; Andrew, M.K.; McNeil, S.A.; Ruiz-Palacios, G.M.; Feng, L.; Kyncl, J.; Vanhems, P. Complicated hospitalization due to influenza: results from the Global Hospital Influenza Network for the 2017–2018 season. *BMC infectious diseases* **2020**, *20*, 1-14.
131. Goncalves, M.T.; Mitsue Saruhashi Shimabukuro, P.; Nakamura Hiraki, K.R.; Braz-Silva, P.H.; Giannecchini, S.; Kai-Wang To, K.; Taminato, M.; Borges de Morais, R. Severe acute respiratory syndrome by influenza and factors associated with death in older adults: a population study. *J Infect Dev Ctries* **2023**, *17*, 241-250, doi:10.3855/jidc.16801.

132. Fuentes, Y.V.; Ibanez-Prada, E.D.; Serrano-Mayorga, C.C.; Pfizenmaier, C.G.; Cano, M.; Boada, N.; Rincon, P.; Garcia-Gallo, E.; Duque, S.; Ocampo, A.F.; et al. Prevalence, incidence, and severity associated with viral respiratory tract infections in Colombian adults before the COVID-19 pandemic. *J Infect Public Health* **2022**, *15*, 1381-1387, doi:10.1016/j.jiph.2022.10.015.
133. de Morais, R.B.; Shimabukuro, P.M.S.; Goncalves, T.M.; Hiraki, K.R.N.; Braz-Silva, P.H.; Giannecchini, S.; To, K.K.W.; Barbosa, D.A.; Taminato, M. Factors associated with death due to severe acute respiratory syndrome caused by influenza: Brazilian population study. *J Infect Public Health* **2022**, *15*, 1388-1393, doi:10.1016/j.jiph.2022.10.016.
134. Sansone, N.M.S.; Boschiero, M.N.; Marson, F.A.L. Epidemiologic Profile of Severe Acute Respiratory Infection in Brazil During the COVID-19 Pandemic: An Epidemiological Study. *Front Microbiol* **2022**, *13*, 911036, doi:10.3389/fmicb.2022.911036.
135. Niquini, R.P.; Lana, R.M.; Pacheco, A.G.; Cruz, O.G.; Coelho, F.C.; Carvalho, L.M.; Villela, D.A.M.; Gomes, M.; Bastos, L.S. Description and comparison of demographic characteristics and comorbidities in SARI from COVID-19, SARI from influenza, and the Brazilian general population. *Cad Saude Publica* **2020**, *36*, e00149420, doi:10.1590/0102-311x00149420.
136. Custodio, A.C.D.; Ribas, F.V.; Toledo, L.V.; Carvalho, C.J.; Lima, L.M.; Freitas, B.A.C. Hospitalizations and mortality by severe acute respiratory syndrome: comparison between the pre-pandemic and pandemic periods. *Rev Bras Epidemiol* **2021**, *24*, e210052, doi:10.1590/1980-549720210052.
137. Mendez-Dominguez, N.I.; Bobadilla-Rosado, L.O.; Fajardo-Ruiz, L.S.; Camara-Salazar, A.; Gomez-Carro, S. Influenza in Yucatan in 2018: Chronology, characteristics and outcomes of ambulatory and hospitalized patients. *Braz J Infect Dis* **2019**, *23*, 358-362, doi:10.1016/j.bjid.2019.08.009.
138. Cooke, B.; Obed, M.; Romandetta, A.; Spinelli, N.; Alzogaray, M.F.; Chaparro, G.; Galeano, L.; Alcorta, M.B.; Balbuena, J.; Russo, M. Rol del virus sincicial respiratorio en una cohorte de adultos mayores. *Actualizaciones en Sida e Infectología* **2019**.
139. Chard, A.N.; Nogareda, F.; Regan, A.K.; Barraza, M.F.O.; Fasce, R.A.; Vergara, N.; Avendaño, M.; Penayo, E.; Vázquez, C.; Von Horoch, M. End-of-season influenza vaccine effectiveness during the Southern Hemisphere 2022 influenza season–Chile, Paraguay, and Uruguay. *International Journal of Infectious Diseases* **2023**, *134*, 39-44.
140. Falcón, A.O.; Rosell Simón, D. Identificación de agentes virales en niños con infección respiratoria aguda. *Revista Cubana de Pediatría* **2022**, *94*.
141. Regan, A.K.; Arriola, C.S.; Couto, P.; Duca, L.; Loayza, S.; Nogareda, F.; de Almeida, W.A.F.; Antman, J.; Araya, S.; Vigueras, M.A.A. Severity of influenza illness by seasonal influenza vaccination status among hospitalised patients in four South American countries, 2013–19: a surveillance-based cohort study. *The Lancet Infectious Diseases* **2023**, *23*, 222-232.
142. Lucion, M.F.; Juárez, M.; Pejit, M.N.; Orqueda, A.S.; Bollón, L.R.; Mistchenko, A.S.; Gentile, Á. Impacto del COVID-19 en la circulación de virus respiratorios en un hospital pediátrico: una ausencia esperada. *Archivos argentinos de pediatría* **2022**, *120*, 6-6.
143. Barraza, M.F.O. Influenza incidence and vaccine effectiveness during the Southern Hemisphere Influenza season—Chile, 2022. *MMWR. Morbidity and Mortality Weekly Report* **2022**, *71*.
144. Bisso, I.C.; Prado, E.; Cantos, J.; Massó, A.; Staneloni, I.; San Román, E.; Huespe, I.; Las Heras, M. Influenza season 2019. Analysis of 143 hospitalized patients. *MEDICINA (Buenos Aires)* **2021**, *81*, 389-395.
145. Mohamed, A.M.; Al Sayyad, A.; Matar, E.; Isa, H.M.; Hasan, W.F.; Hashim, N.; Alajaimi, B.A.; Aldolabi, Q. Factors associated with poor outcomes in patients with severe acute respiratory infections in Bahrain. *Influenza Other Respir Viruses* **2023**, *17*, e13133, doi:10.1111/irv.13133.

146. Almojali, A.I.; Alshareef, M.S.; Aljadoa, O.F.; Alotaibi, F.F.; Masuadi, E.M.; Hameed, T.K. The prevalence of serious bacterial infections in infants 90 days and younger with viral respiratory tract infections. *Saudi Med J* **2022**, *43*, 1007-1012, doi:10.15537/smj.2022.43.9.20220400.
147. Regragui, Z.; Bimouhen, A.; El Falaki, F.; Ihazmad, H.; Benkerroum, S.; Triki, S.; Cherkaoui, I.; Mahraoui, C.; Filali-Maltouf, A.; Medraoui, L.; et al. Influenza-associated severe acute respiratory infections among children under five years old in Morocco, September 2017 to March 2019. *Infez Med* **2022**, *30*, 446-453, doi:10.53854/liim-3003-14.
148. Shafiekhani, M.; Kazemi, K.; Bahador, A.; Imanieh, M.H.; Karimzadeh, P. Pediatric liver and kidney transplantation in the era of COVID-19: a follow-up study from a tertiary referral center in Iran. *BMC Surg* **2021**, *21*, 240, doi:10.1186/s12893-021-01226-y.
149. Alsayed, S.M.; Alandijany, T.A.; El-Kafrawy, S.A.; Hassan, A.M.; Bajrai, L.H.; Faizo, A.A.; Mulla, E.A.; Aljahdali, L.S.; Alquthami, K.M.; Zumla, A.; et al. Pattern of Respiratory Viruses among Pilgrims during 2019 Hajj Season Who Sought Healthcare Due to Severe Respiratory Symptoms. *Pathogens* **2021**, *10*, doi:10.3390/pathogens10030315.
150. Mohebi, L.; Karami, H.; Mirsalehi, N.; Ardestani, N.H.; Yavarian, J.; Mard-Soltani, M.; Mokhatri-Azad, T.; Salimi, V. A delayed resurgence of respiratory syncytial virus (RSV) during the COVID-19 pandemic: An unpredictable outbreak in a small proportion of children in the Southwest of Iran, April 2022. *J Med Virol* **2022**, *94*, 5802-5807, doi:10.1002/jmv.28065.
151. Alosaimi, B.; Naeem, A.; Hamed, M.E.; Alkadi, H.S.; Alanazi, T.; Al Rehily, S.S.; Almutairi, A.Z.; Zafar, A. Influenza co-infection associated with severity and mortality in COVID-19 patients. *Virol J* **2021**, *18*, 127, doi:10.1186/s12985-021-01594-0.
152. Dawood, H.; Mahdi, S.G. A retrospective study of demographic profile and outcome of Sever acute respiratory illness (SARI) in Iraq. *Journal of the Faculty of Medicine Baghdad* **2021**, *63*.
153. Tavanaee-Sani, A.; Sharifi, R.; Nehbandani, Z.; Masoudi, M.-H.; Solouki, Y. Epidemiology and Clinical Outcomes of Patients with Confirmed Influenza in Mashhad, Iran in 2019. *International Journal of Infection* **2021**, *8*.
154. Daneshyar, Z.; Goli, H.R.; Mirzaei, B.; Rabie Rudsari, M.; Haghshenas, M. Prevalence and Clinical Symptoms of Human Parainfluenza and Influenza Infections in Patients Admitted to Mazandaran Province Health Centers in 2019. Research in Molecular Medicine. 2019; 7 (3): 29-38. **1974**.
155. Zendehrouh, M.; Karimi, A.; Azimi, L. Respiratory viral infections among children hospitalized in a great Referral Hospital in Iran during the Coronavirus pandemic. *Archives of Pediatric Infectious Diseases* **2023**, *11*.
156. Le, Y.H.; Nguyen, K.C.; Coleman, K.K.; Nguyen, T.T.; Than, S.T.; Phan, H.H.; Nguyen, M.D.; Ngu, N.D.; Phan, D.T.; Hoang, P.V.M.; et al. Virus detections among patients with severe acute respiratory illness, Northern Vietnam. *PLoS One* **2020**, *15*, e0233117, doi:10.1371/journal.pone.0233117.
157. Kim, K.J.; Yun, S.G.; Cho, Y.; Nam, M.H.; Lee, C.K. Usefulness of Combining Sputum and Nasopharyngeal Samples for Viral Detection by Reverse Transcriptase PCR in Adults Hospitalized with Acute Respiratory Illness. *Microbiol Spectr* **2022**, *10*, e0277522, doi:10.1128/spectrum.02775-22.
158. Choi, M.J.; Yun, J.W.; Song, J.Y.; Ko, K.; Mould, J.F.; Cheong, H.J. A Comparative Analysis of Influenza-Associated Disease Burden with Different Influenza Vaccination Strategies for the Elderly Population in South Korea. *Vaccines (Basel)* **2022**, *10*, doi:10.3390/vaccines10091387.
159. Boonnak, K.; Mansanguan, C.; Schuerch, D.; Boonyuen, U.; Lerdsamran, H.; Jiamsomboon, K.; Sae Wang, F.; Huntrup, A.; Prasertsopon, J.; Kosoltanapiwat, N.; et al. Molecular Characterization of Seasonal Influenza A and B from Hospitalized Patients in Thailand in 2018-2019. *Viruses* **2021**, *13*, doi:10.3390/v13060977.

160. Kirca, F.; Aydogan, S.; Gozalan, A.; Guler, E.; Uyan Erten, A.Z.; Ozsen Uygur, A.S.; Dogan, A.; Dinc, B. Impact of non-pharmaceutical interventions on circulating respiratory viruses during the COVID-19 pandemic in Turkey. *Ann Saudi Med* **2023**, *43*, 143-153, doi:10.5144/0256-4947.2023.143.
161. Kurskaya, O.G.; Prokopyeva, E.A.; Sobolev, I.A.; Solomatina, M.V.; Saroyan, T.A.; Dubovitskiy, N.A.; Derko, A.A.; Nokhova, A.R.; Anoshina, A.V.; Leonova, N.V.; et al. Changes in the Etiology of Acute Respiratory Infections among Children in Novosibirsk, Russia, between 2019 and 2022: The Impact of the SARS-CoV-2 Virus. *Viruses* **2023**, *15*, doi:10.3390/v15040934.
162. Agca, H.; Akalin, H.; Saglik, I.; Hacimustafaoglu, M.; Celebi, S.; Ener, B. Changing epidemiology of influenza and other respiratory viruses in the first year of COVID-19 pandemic. *J Infect Public Health* **2021**, *14*, 1186-1190, doi:10.1016/j.jiph.2021.08.004.
163. Gaur, B.; Saha, S.; Iuliano, A.D.; Rai, S.K.; Krishnan, A.; Jain, S.; Whitaker, B.; Winchell, J.; Lal, R.B.; Broor, S. Use of TaqMan Array card for the detection of respiratory viral pathogens in children under 5 years old hospitalised with acute medical illness in Ballabgarh, Haryana, India. *Indian J Med Microbiol* **2019**, *37*, 105-108, doi:10.4103/ijmm.IJMM_18_146.
164. Motlogeloa, O.; Fitchett, J.M.; Sweijd, N. Defining the South African Acute Respiratory Infectious Disease Season. *Int J Environ Res Public Health* **2023**, *20*, doi:10.3390/ijerph20021074.
165. Chiu, N.-C.; Chi, H.; Tai, Y.-L.; Peng, C.-C.; Tseng, C.-Y.; Chen, C.-C.; Tan, B.F.; Lin, C.-Y. Impact of wearing masks, hand hygiene, and social distancing on influenza, enterovirus, and all-cause pneumonia during the coronavirus pandemic: retrospective national epidemiological surveillance study. *Journal of medical Internet research* **2020**, *22*, e21257.
166. Quéromès, G.; Frobert, E.; Burtseva, E.; Drăgănescu, A.; Koul, P.A.; Komissarov, A.; Laguna-Torres, V.A.; Leblanc, J.; López-Labrador, F.X.; Medić, S.; et al. Clinical and phylogenetic influenza dynamics for the 2019-20 season in the global influenza hospital surveillance network (GIHSN) – Pilot study. *Journal of Clinical Virology* **2022**, *152*, 105184, doi:https://doi.org/10.1016/j.jcv.2022.105184.
167. Pang, Y.K.; Ismail, A.I.; Chan, Y.F.; Cheong, A.; Chong, Y.M.; Doshi, P.; Lau, J.Z.H.; Khor, J.; Wang, L.P.L.; Leong, C.L.; et al. Influenza in Malaysian adult patients hospitalized with community-acquired pneumonia, acute exacerbation of chronic obstructive pulmonary disease or asthma: a multicenter, active surveillance study. *BMC Infectious Diseases* **2021**, *21*, 644, doi:10.1186/s12879-021-06360-9.
168. P, S.; Dhandapani, N.S. Evaluation of Pregnancy, Younger Age, and Old Age as Independent Risk Factors for Poor Hospitalization Outcomes in Influenza A (H1N1)pdm09 Virus a Decade After the Pandemic. *Cureus* **2020**, *12*, e11762, doi:10.7759/cureus.11762.
169. Hwang, S.-H.; Lee, H.; Jung, M.; Kim, S.-H.; Sung, H.K.; Oh, M.-d.; Lee, J.Y. Incidence, Severity, and Mortality of Influenza During 2010–2020 in Korea: A Nationwide Study Based on the Population-Based National Health Insurance Service Database. *J Korean Med Sci* **2023**, *38*.
170. Hong, T.H.; Lee, H.S.; Kim, N.-E.; Lee, K.J.; Kim, Y.K.; An, J.N.; Kim, J.-H.; Kim, H.W.; Park, S. Recent increases in influenza-related hospitalizations, critical care resource use, and In-hospital mortality: a 10-year population-based study in South Korea. *Journal of Clinical Medicine* **2022**, *11*, 4911.
171. Hegde, S.; Thyagaraj, V.; Prabhu, D. "H1N1 influenza revisited: Our experience of the 2019 outbreak". *Bangladesh Journal of Medical Science* **2022**, *21*, 426-431, doi:10.3329/bjms.v21i2.58077.
172. Chen, A.P.-L.; Chuang, C.; Huang, Y.-C.; Wu, P.-F.; Huang, S.-F.; Cheng, N.-C.; Lin, Y.-T.; Chen, S.-J.; Huang, L.-J.; Lee, C.-L.; et al. The epidemiology and etiologies of respiratory tract infection in Northern Taiwan during the early phase of coronavirus disease 2019 (COVID-19) outbreak. *Journal of Microbiology, Immunology and Infection* **2021**, *54*, 801-807, doi:https://doi.org/10.1016/j.jmii.2021.05.006.

173. C, T.; Dv, S.; A, S.; V, K.; A, K. A Concurrent Comparison of the Epidemiology and Clinical Presentation of Patients Hospitalized with Pandemic 2009 (H1N1) Influenza and Seasonal Influenza-A in Sub-himalayan Region of Himachal Pradesh. *J Assoc Physicians India* **2019**, *67*, 70-74.
174. Murillo-Zamora, E.; Mendoza-Cano, O.; Delgado-Enciso, I.; Guzmán-Esquivel, J. National retrospective cohort study to identify risk factors for in-hospital 30-day lethality in laboratory-confirmed cases of influenza. *Rev Clin Esp* **2021**, *221*, 76-85, doi:10.1016/j.rce.2020.05.016.
175. de Morais, R.B.; Shimabukuro, P.M.S.; Gonçalves, T.M.; Hiraki, K.R.N.; Braz-Silva, P.H.; Giannecchini, S.; To, K.K.W.; Barbosa, D.A.; Taminato, M. Factors associated with death due to severe acute respiratory syndrome caused by influenza: Brazilian population study. *J Infect Public Health* **2022**, *15*, 1388-1393, doi:10.1016/j.jiph.2022.10.016.
176. Martins Gonçalves, T.; Mitsue Saruhashi Shimabukuro, P.; Nakamura Hiraki, K.R.; Braz-Silva, P.H.; Giannecchini, S.; Kai-Wang To, K.; Taminato, M.; Borges de Morais, R. Severe acute respiratory syndrome by influenza and factors associated with death in older adults: a population study. *The Journal of Infection in Developing Countries* **2023**, *17*, 241-250, doi:10.3855/jidc.16801.
177. Regan, A.K.; Arriola, C.S.; Couto, P.; Duca, L.; Loayza, S.; Nogareda, F.; de Almeida, W.A.F.; Antman, J.; Araya, S.; Avendaño Vigueras, M.A.; et al. Severity of influenza illness by seasonal influenza vaccination status among hospitalised patients in four South American countries, 2013–19: a surveillance-based cohort study. *The Lancet Infectious Diseases* **2023**, *23*, 222-232, doi:10.1016/S1473-3099(22)00493-5.
178. Carboni Bisso, I.; Prado, E.; Cantos, J.; Massó, A.; Staneloni, I.; San Román, E.; Huespe, I.; Las Heras, M. Influenza season 2019. Analysis of 143 hospitalized patients. *Medicina (B Aires)* **2021**, *81*, 389-395.
179. Moreira, A.L.E.; da Silva, P.A.N.; Assunção, L.D.P.; Santos, M.O.; Ito, C.R.M.; de Araújo, K.M.; Cunha, M.O.; Rabelo, V.D.C.; de Souza, P.P.; Maia, S.B.S.; et al. Profile analysis of emerging respiratory virus in children. *Eur J Clin Microbiol Infect Dis* **2023**, *42*, 873-882, doi:10.1007/s10096-023-04615-8.
180. Nesselroth, D.; Yakub Hana, H.; Gleyzer, A.; Simoes, E.A.F.; Abu Atta, M.; Ben Yehuda, Y.; Bibi, H.; Somekh, I.; Somekh, E. Comparison of the medical burden of COVID-19 with seasonal influenza and measles outbreaks. *Acta Paediatr* **2022**, *111*, 595-601, doi:10.1111/apa.16210.
181. Jurkowicz, M.; Nemet, I.; Atari, N.; Fratty, I.S.; Kliker, L.; Sherbany, H.; Keller, N.; Leibovitz, E.; Mendelson, E.; Mandelboim, M.; et al. Cocirculation of A(H3N2) and B/Victoria increased morbidity in hospitalized patients in the 2019-2020 A(H1N1)pdm09 predominant influenza season in Israel. *J Med Virol* **2023**, *95*, e28498, doi:10.1002/jmv.28498.
182. Chan, K.S.; Liang, F.W.; Tang, H.J.; Toh, H.S.; Yu, W.L. Collateral benefits on other respiratory infections during fighting COVID-19. *Med Clin (Barc)* **2020**, *155*, 249-253, doi:10.1016/j.medcli.2020.05.026.
183. Martins-Filho, P.R.; Júnior, J.M.O.; Santos, C.A.D. Case-fatality rates and risk of death from COVID-19 and influenza A/H3N2 in Brazil: A nationwide ecological study. *Enferm Infecc Microbiol Clin (Engl Ed)* **2023**, *41*, 199-201, doi:10.1016/j.eimce.2022.05.017.
184. Ríos-Silva, M.; Trujillo, X.; Huerta, M.; Benites-Godínez, V.; Guzmán-Esquivel, J.; Bricio-Barrios, J.A.; Mendoza-Cano, O.; Lugo-Radillo, A.; Murillo-Zamora, E. Reemerging Influenza Virus Infections during the Dominance of the Omicron SARS-CoV-2 Variant in Mexico. *Pathogens* **2022**, *11*, doi:10.3390/pathogens11101181.
185. Hashemi, S.A.; Safamanesh, S.; Ghasemzadeh-Moghaddam, H.; Ghafouri, M.; Azimian, A. High prevalence of SARS-CoV-2 and influenza A virus (H1N1) coinfection in dead patients in Northeastern Iran. *J Med Virol* **2021**, *93*, 1008-1012, doi:10.1002/jmv.26364.

186. Tavanaee-Sani, A.; Sharifi, R.; Nehbandani, Z.; Masoudi, M.; Solouki, Y. Epidemiology and Clinical Outcomes of Patients with Confirmed Influenza in Mashhad, Iran in 2019. *Int J Infect* **2021**, *8*, e116795, doi:10.5812/iji.116795.
187. Daneshyar, z.; Goli, H.r.; Mirzaei, B.; Rabie, M.; Haghshenas, M.r. Prevalence and clinical symptoms of Human Parainfluenza and Influenza infections in patients admitted to Mazandaran province health centers, 2019. *Research in Molecular Medicine* **2019**, *7*, 29-38, doi:https://doi.org/10.32598/rmm.7.3.29.
188. Choreño-Parra, J.A.; Jiménez-Álvarez, L.A.; Ramírez-Martínez, G.; Sandoval-Vega, M.; Salinas-Lara, C.; Sánchez-Garibay, C.; Luna-Rivero, C.; Hernández-Montiel, E.M.; Fernández-López, L.A.; Cabrera-Cornejo, M.F.; et al. CXCL17 Is a Specific Diagnostic Biomarker for Severe Pandemic Influenza A(H1N1) That Predicts Poor Clinical Outcome. *Front Immunol* **2021**, *12*, 633297, doi:10.3389/fimmu.2021.633297.
189. Lucas, P.C.C.; Lorenz, C.; Florez-Montero, G.L.; Palasio, R.G.S.; Portella, T.P.; Monteiro, P.C.M.; Yu, A.L.F.; Carvalhanas, T. Institutional outbreaks of influenza-like illnesses in the state of São Paulo: an analysis of the epidemiological profile during the COVID-19 pandemic. *Public Health* **2023**, *221*, 142-149, doi:10.1016/j.puhe.2023.06.018.
190. Fuentes, Y.V.; Ibáñez-Prada, E.D.; Serrano-Mayorga, C.C.; Pfizenmaier, C.G.; Cano, M.; Boada, N.; Rincon, P.; García-Gallo, E.; Duque, S.; Ocampo, A.F.; et al. Prevalence, incidence, and severity associated with viral respiratory tract infections in Colombian adults before the COVID-19 pandemic. *J Infect Public Health* **2022**, *15*, 1381-1387, doi:10.1016/j.jiph.2022.10.015.
191. Saraceni, V.; Cruz, O.G.; Cavalcante, J.R.; Vieira, F.; Cardoso, B.B.; Cruz, D.; Aguilar, G.M.O.; Durovni, B.; Soranz, D.; Garcia, M.H.O. Excess mortality from all causes during the COVID-19 pandemic in the city of Rio de Janeiro, Brazil. *Rev Bras Epidemiol* **2023**, *26*, e230013, doi:10.1590/1980-549720230013.
192. Tapia-Conyer, R.; Betancourt-Cravioto, M.; Montoya, A.; Falcon-Lezama, J.A.; Alfaro-Cortes, M.M.; Saucedo-Martinez, R. A Call for a Reform of the Influenza Immunization Program in Mexico: Epidemiologic and Economic Evidence for Decision Making. *Vaccines (Basel)* **2021**, *9*, doi:10.3390/vaccines9030286.
193. Wesley, M.G.; Tinoco, Y.; Patel, A.; Suntarratiwong, P.; Hunt, D.; Sinthuwattanawibool, C.; Soto, G.; Kittikraisak, W.; Das, P.K.; Arriola, C.S.; et al. Performance of Symptom-Based Case Definitions to Identify Influenza Virus Infection Among Pregnant Women in Middle-Income Countries: Findings From the Pregnancy and Influenza Multinational Epidemiologic (PRIME) Study. *Clin Infect Dis* **2021**, *73*, e4321-e4328, doi:10.1093/cid/ciaa1697.
194. Warrell, C.E.; Phyo, A.P.; Win, M.M.; McLean, A.R.D.; Watthanaworawit, W.; Swe, M.M.M.; Soe, K.; Lin, H.N.; Aung, Y.Y.; Ko, C.K.; et al. Observational study of adult respiratory infections in primary care clinics in Myanmar: understanding the burden of melioidosis, tuberculosis and other infections not covered by empirical treatment regimes. *Trans R Soc Trop Med Hyg* **2021**, *115*, 914-921, doi:10.1093/trstmh/trab024.
195. Mwakibete, L.; Takahashi, S.; Ahyong, V.; Black, A.; Rek, J.; Ssewanyana, I.; Kamya, M.; Dorsey, G.; Jagannathan, P.; Rodriguez-Barraquer, I.; et al. Metagenomic next-generation sequencing to characterize potential etiologies of non-malarial fever in a cohort living in a high malaria burden area of Uganda. *PLOS Glob Public Health* **2023**, *3*, e0001675, doi:10.1371/journal.pgph.0001675.
196. Jarju, S.; Greenhalgh, K.; Wathuo, M.; Banda, M.; Camara, B.; Mendy, S.; Sowe, G.; Dahaba, P.O.; Jammeh, L.; Bajinka, Y.; et al. Viral Etiology, Clinical Features and Antibiotic Use in Children <5 Years of Age in the Gambia Presenting With Influenza-like Illness. *Pediatr Infect Dis J* **2020**, *39*, 925-930, doi:10.1097/INF.0000000000002761.

197. Ntagereka, P.B.; Basengere, R.A.; Baharanyi, T.C.; Kashosi, T.M.; Buhendwa, J.C.; Bisimwa, P.B.; Kusinza, A.B.; Mugumaarhahama, Y.; Shukuru, D.W.; Patrick, S.B.; et al. Molecular Evidence of Coinfection with Acute Respiratory Viruses and High Prevalence of SARS-CoV-2 among Patients Presenting Flu-Like Illness in Bukavu City, Democratic Republic of Congo. *Can J Infect Dis Med Microbiol* **2022**, *2022*, 1553266, doi:10.1155/2022/1553266.
198. Miring'u, G.; Muriithi, B.; Shoji, H.; Symekher, S.M.L.; Wandera, E.A.; Majisu, C.; Takei, M.; Mwiraria, K.; Saito, Y.; Kaneko, S.; et al. Characterization of influenza infection in a high-income urban setting in Nairobi, Kenya. *Trop Med Health* **2022**, *50*, 69, doi:10.1186/s41182-022-00463-y.
199. Suryadevara, M.; Fajardo, F.P.; Aponte, C.C.; Carrillo Aponte, J.L.; Prado, E.O.; Hidalgo, I.; Bonville, C.A.; Torres, I.; Domachowske, J.B. Etiologies of outpatient medically attended acute respiratory infections among young Ecuadorian children prior to the start of the 2020 SARS-CoV-2 pandemic. *Influenza Other Respir Viruses* **2023**, *17*, e13056, doi:10.1111/irv.13056.
200. Gomez de la Torre Pretell, J.C.; Hueda-Zavaleta, M.; Caceres-DelAguila, J.A.; Barletta-Carrillo, C.; Copaja-Corzo, C.; Poccorpachi, M.; Delgado, M.S.V.; Sanchez, G.; Benites-Zapata, V.A. Clinical Characteristics Associated with Detected Respiratory Microorganism Employing Multiplex Nested PCR in Patients with Presumptive COVID-19 but Negative Molecular Results in Lima, Peru. *Trop Med Infect Dis* **2022**, *7*, doi:10.3390/tropicalmed7110340.
201. Kandeel, A.; Fahim, M.; Deghedy, O.; Roshdy, W.H.; Khalifa, M.K.; Shesheny, R.E.; Kandeil, A.; Naguib, A.; Afifi, S.; Mohsen, A.; et al. Resurgence of influenza and respiratory syncytial virus in Egypt following two years of decline during the COVID-19 pandemic: outpatient clinic survey of infants and children, October 2022. *BMC Public Health* **2023**, *23*, 1067, doi:10.1186/s12889-023-15880-9.
202. Hindupur, A.; Dhandapani, P.; Menon, T. Influenza Virus Among Children with Acute Respiratory Infections in Chennai, India. *Indian Pediatrics* **2019**, *56*, 74-75.
203. Kang, J.M.; Jung, J.; Kim, Y.E.; Huh, K.; Hong, J.; Kim, D.W.; Kim, M.Y.; Jung, S.Y.; Kim, J.H.; Ahn, J.G. Temporal Correlation Between Kawasaki Disease and Infectious Diseases in South Korea. *JAMA Netw Open* **2022**, *5*, e2147363, doi:10.1001/jamanetworkopen.2021.47363.
204. Kim, S.; Park, J.O.; Lee, H.A.; Park, H.A.; Lee, C.A.; Wang, S.J.; Jung, E.J. Unintended beneficial effects of COVID-19 on influenza-associated emergency department use in Korea. *Am J Emerg Med* **2022**, *59*, 1-8, doi:10.1016/j.ajem.2022.06.039.
205. Thomas, S.; Emara, M.M.; Ouhtit, A.; Nader, J.D.; Nasrallah, G.K.; Coyle, P.V.; Althani, A.A.; Al Maslamani, M.A.; Yassine, H.M. Influenza prevalence and vaccine efficacy among diabetic patients in Qatar. *J Infect Public Health* **2023**, *16*, 808-815, doi:10.1016/j.jiph.2023.03.011.
206. Khasawneh, A.I.; Himsawi, N.M.; Abu-Raideh, J.A.; Sammour, A.; Abu Safieh, H.; Obeidat, A.; Azab, M.; Tarifi, A.A.; Al Khawaldeh, A.; Al-Momani, H.; et al. Prevalence of SARS-COV-2 and other respiratory pathogens among a Jordanian subpopulation during Delta-to-Omicron transition: Winter 2021/2022. *PLoS One* **2023**, *18*, e0283804, doi:10.1371/journal.pone.0283804.
207. Taiwan centers for disease control. Taiwan Weekly Flu News. **2019**.
208. Taiwan centers for disease control. Taiwan Weekly Flu News. **2020**.
209. Taiwan centers for disease control. Taiwan Weekly Flu News. **2023**.
210. Izu, A.; Nunes, M.C.; Solomon, F.; Baillie, V.; Serafin, N.; Verwey, C.; Moore, D.P.; Laubscher, M.; Ncube, M.; Olwagen, C.; et al. All-cause and pathogen-specific lower respiratory tract infection hospital admissions in children younger than 5 years during the COVID-19 pandemic (2020-22) compared with the pre-pandemic period (2015-19) in South Africa: an observational study. *Lancet Infect Dis* **2023**, *23*, 1031-1041, doi:10.1016/s1473-3099(23)00200-1.

211. Barraza, M.F.O.; Fasce, R.A.; Nogareda, F.; Marcenac, P.; Mallegas, N.V.; Alister, P.B.; Loayza, S.; Chard, A.N.; Arriola, C.S.; Couto, P.; et al. Influenza incidence and vaccine effectiveness during the Southern Hemisphere Influenza season-Chile, 2022. *Am J Transplant* **2022**, *22*, 3170-3174, doi:10.1111/ajt.16685.
212. Sitthikarnkha, P.; Uppala, R.; Niamsanit, S.; Sutra, S.; Thepsuthammarat, K.; Techasatian, L.; Teeratakulpisarn, J. Epidemiology of acute lower respiratory tract infection hospitalizations in Thai children: A 5-year national data analysis. *Influenza Other Respir Viruses* **2022**, *16*, 142-150, doi:10.1111/irv.12911.
213. Danilenko, A.V.; Kolosova, N.P.; Shvalov, A.N.; Ilyicheva, T.N.; Svyatchenko, S.V.; Durymanov, A.G.; Bulanovich, J.A.; Goncharova, N.I.; Susloparov, I.M.; Marchenko, V.Y.; et al. Evaluation of HA-D222G/N polymorphism using targeted NGS analysis in A(H1N1)pdm09 influenza virus in Russia in 2018-2019. *PLoS One* **2021**, *16*, e0251019, doi:10.1371/journal.pone.0251019.
214. Naomi Adeline, J.B., Agnes Chetty, Susan Fock-Tave, Annalisa Labiche, Sanjeev Pugazhendhi, Emelyn Shroff, Bernard Valentin, Williams Ituen-Umanah *Health of our Nation, Annual Health Sector Performance Report, 2018*; Ministry of Health 2019.
215. LoevinsohnG., M.T., Sinywimaanzi P., et al. Influenza and RSV infection in rural Zambia. **2020**.
216. Pachas, P.; Donaires, F.; Gavilán, R.G.; Quino, W.; Vidal, M.; Cabezas, C.; García, M.; Huaringa, M.; Peceros, F.; Valdivia, F.; et al. Infectious agents in biological samples from patients with Guillain-Barré syndrome in Peru, 2018-2019. *Rev Peru Med Exp Salud Publica* **2020**, *37*, 681-688, doi:10.17843/rpmesp.2020.374.5169.
217. Ghosh, P.; Chaudhury, N.; Nath, A.; Mukherjee, N. Molecular Detection of H1N1 and Impact of Cytokines among Infected Patients with Respiratory Distress: A Cross-sectional Study. **2023**, *17*, 24-29, doi:10.7860/JCDR/2023/61070.17545.
218. Ali, S.A.; Jabeen, K.; Farooqi, J.; Niamatullah, H.; Siddiqui, A.F.; Awan, S.; Akbar, A.; Irfan, M. Invasive pulmonary aspergillosis in critically ill patients with pneumonia due to COVID-19, influenza, and community-acquired pneumonia: A prospective observational study. *Curr Med Mycol* **2022**, *8*, 16-24, doi:10.18502/cmm.8.2.10328.
219. Nguyen, S.N.; Vu, L.T.; Vu, Q.V.; Tran, T.T.; Dinh, V.T.T. Clinical Epidemiology Characteristics and Etiology of Febrile Neutropenia in Children: Analysis of 421 Cases. *Hematol Rep* **2022**, *14*, 245-252, doi:10.3390/hematolrep14030034.
220. Anas, M.; Ritonga, D. Comparative Demographics of Influenza A by Genetic Subtypes through Surveillance Approach for Severe Acute Respiratory Infection in Children: A Hospital-Based Study from 2019 to 2020. *International Medical Journal (1994)* **2022**, *29*, 1-5.
221. Kamata, K.; Thein, K.N.; Di Ja, L.; Win, N.C.; Win, S.M.K.; Suzuki, Y.; Ito, A.; Osada, H.; Chon, I.; Phyu, W.W.; et al. Clinical manifestations and outcome of viral acute lower respiratory infection in hospitalised children in Myanmar. *BMC Infectious Diseases* **2022**, *22*, 350, doi:10.1186/s12879-022-07342-1.
222. Toh, T.H.; Hii, K.C.; Fieldhouse, J.K.; Ting, J.; Berita, A.; Nguyen, T.T.; Wong, S.C.; Wong, T.M.; Lim, W.H.; Ha, S.J.; et al. High Prevalence of Viral Infections Among Hospitalized Pneumonia Patients in Equatorial Sarawak, Malaysia. *Open Forum Infect Dis* **2019**, *6*, ofz074, doi:10.1093/ofid/ofz074.
223. Byeon, K.H.; Kim, J.; Choi, B.Y.; Kim, J.Y.; Lee, N. Factors Affecting the Incidence of Hospitalized Pneumonia after Influenza Infection in Korea Using the National Health Insurance Research Database, 2014-2018: Focusing on the Effect of Antiviral Therapy in the 2017 Flu Season. *J Korean Med Sci* **2020**, *35*, e318, doi:10.3346/jkms.2020.35.e318.

224. Kang, M.; Sarkar, S.; Angurana, S.K.; Singh, P.; Rana, M.; Bora, I.; Chetanya, R.; Singh, B.; Muralidharan, J.; Ratho, R.K. Paradigm shift of respiratory viruses causing lower respiratory tract infection in children during COVID-19 pandemic in India. *J Infect Dev Ctries* **2023**, *17*, 961-970, doi:10.3855/jidc.17727.
225. Nambafu, J.; Achakolong, M.; Mwendwa, F.; Bwika, J.; Riunga, F.; Gitau, S.; Patel, H.; Adam, R.D. A prospective observational study of community acquired pneumonia in Kenya: the role of viral pathogens. *BMC Infect Dis* **2021**, *21*, 703, doi:10.1186/s12879-021-06388-x.
226. Wadilo, F.; Feleke, A.; Gebre, M.; Mihret, W.; Seyoum, T.; Melaku, K.; Howe, R.; Mulu, A.; Mihret, A. Viral etiologies of lower respiratory tract infections in children < 5 years of age in Addis Ababa, Ethiopia: a prospective case-control study. *Virol J* **2023**, *20*, 163, doi:10.1186/s12985-023-02131-x.
227. Dagan, R.; van der Beek, B.A.; Ben-Shimol, S.; Greenberg, D.; Shemer-Avni, Y.; Weinberger, D.M.; Danino, D. The COVID-19 pandemic as an opportunity for unravelling the causative association between respiratory viruses and pneumococcus-associated disease in young children: a prospective study. *EBioMedicine* **2023**, *90*, 104493, doi:10.1016/j.ebiom.2023.104493.
228. Shafran, N.; Shafran, I.; Ben-Zvi, H.; Sofer, S.; Sheena, L.; Krause, I.; Shlomai, A.; Goldberg, E.; Sklan, E.H. Secondary bacterial infection in COVID-19 patients is a stronger predictor for death compared to influenza patients. *Sci Rep* **2021**, *11*, 12703, doi:10.1038/s41598-021-92220-0.
229. Al-Zayadneh, E.; Mohammad Abu Assab, D.; Adeeb Arabiat, E.; Al-Iede, M.; Ahmad Kayed, H.; Daher, A. The burden of influenza and other respiratory viruses in hospitalized infants and children in a university hospital, Jordan. *Multidiscip Respir Med* **2021**, *16*, 763, doi:10.4081/mrm.2021.763.
230. Kamata, K.; Thein, K.N.; Di Ja, L.; Win, N.C.; Win, S.M.K.; Suzuki, Y.; Ito, A.; Osada, H.; Chon, I.; Phyu, W.W.; et al. Clinical manifestations and outcome of viral acute lower respiratory infection in hospitalised children in Myanmar. *BMC Infect Dis* **2022**, *22*, 350, doi:10.1186/s12879-022-07342-1.
231. Madhuravasal Krishnan, J.; Jayaraman, D.; Kancharla, A.; Thangam, A.; Venkatramanan, P.; Scott, J.X. Role of Polymerase Chain Reaction-Based Diagnosis of Respiratory Viruses in Febrile Neutropenic Patients. *Cureus* **2023**, *15*, e33314, doi:10.7759/cureus.33314.
232. Ahuja, N.; Gorain, S.; Pal, P.; Das, M. Viral Aetiology of Severe Acute Lower Respiratory Tract Infection in Children from the Paediatric Intensive Care Unit at a Tertiary Care Hospital, Eastern India- A Retrospective Study. *JOURNAL OF CLINICAL AND DIAGNOSTIC RESEARCH* **2022**, *16*, doi:10.7860/JCDR/2022/57925.17026.
233. Suman, S.V.B., Kumar D. A Hospital Based Observational Study to Assess the Clinic-Etiological Profile and Prognostic Indicators in Critically Ill Patients who Develop Acute Respiratory Distress Syndrome (ARDS) in Medical Intensive Care Unit. *European journal of molecular & clinical medicine* **2022**, *9*.
234. Gómez de la Torre Pretell, J.C.; Hueda-Zavaleta, M.; Cáceres-DelAguila, J.A.; Barletta-Carrillo, C.; Copaja-Corzo, C.; Poccorpachi, M.; Delgado, M.S.V.; Sanchez, G.; Benites-Zapata, V.A. Clinical Characteristics Associated with Detected Respiratory Microorganism Employing Multiplex Nested PCR in Patients with Presumptive COVID-19 but Negative Molecular Results in Lima, Peru. *Trop Med Infect Dis* **2022**, *7*, doi:10.3390/tropicalmed7110340.
235. Naga, I.S.; Elsawaf, G.E.; Elzalabany, M.; Eltalkhawy, M.Y.; Kader, O. Human coronavirus OC43 and other respiratory viruses from acute respiratory infections of Egyptian children. *Acta Microbiol Immunol Hung* **2020**, *67*, 112-119, doi:10.1556/030.2020.01059.

236. Duarte, F.G.; Barberino, M.G.; da Silva Moreira, S.; Reis, J.N.; Spinardi, J.R.; de Almeida, R.S.; Allen, K.E.; Alexander-Parrish, R.; Brim, R.; de Araujo Neto, C.A.; et al. Incidence, aetiology and serotype coverage for pneumococcal vaccines of community-acquired pneumonia in adults: a population-based prospective active surveillance study in Brazil. *BMJ Open* **2022**, *12*, e059824, doi:10.1136/bmjopen-2021-059824.
237. Trivedi, A.; Fontelera, M.; Lai, A. SARS-COV-2 Screening of Health Care Workers in Brunei Darussalam. *Workplace Health Saf* **2022**, *70*, 452-458, doi:10.1177/21650799211062802.
238. Gupta, V.; Banavara Rajanna, L.; Upadhyay, K.; Bhatia, R.; Madhav Reddy, N.; Malik, D.; Srivastava, A. Olfactory and Gustatory Dysfunction in COVID-19 Patients from Northern India: A Cross-Sectional Observational Study. *Indian J Otolaryngol Head Neck Surg* **2021**, *73*, 218-225, doi:10.1007/s12070-021-02391-5.
239. Khan, M.S.; Haq, I.; Qurieshi, M.A.; Majid, S.; Bhat, A.A.; Qazi, T.B.; Chowdri, I.N.; Sabah, I.; Kawoosa, M.F.; Lone, A.A.; et al. SARS-CoV-2 Seroprevalence Among Healthcare Workers by Workplace Exposure Risk in Kashmir, India. *J Hosp Med* **2021**, *16*, 274-281, doi:10.12788/jhm.3609.
240. Knust, B.; Wongjindanon, N.; Moe, A.A.; Herath, L.; Kaloy, W.; Soe, T.T.; Sataranon, P.; Oo, H.M.; Myat, K.Z.; Win, Z.; et al. Enhancing Respiratory Disease Surveillance to Detect COVID-19 in Shelters for Displaced Persons, Thailand-Myanmar Border, 2020-2021. *Emerg Infect Dis* **2022**, *28*, S17-S25, doi:10.3201/eid2813.220324.
241. Goni, M.D.; Hasan, H.; Naing, N.N.; Wan-Arfah, N.; Deris, Z.Z.; Arifin, W.N.; Baaba, A.A. Impact of a Health Education Intervention on the Incidence of Influenza-Like Illnesses (ILI) During Hajj via Smartphone Application. *J Immigr Minor Health* **2023**, *25*, 870-881, doi:10.1007/s10903-022-01443-4.
242. World Health Organization. ILI surveillance outputs: WHO Global Influenza Program. Available online: https://www.who.int/teams/global-influenza-programme/surveillance-and-monitoring/influenza-surveillance-outputs (accessed on
243. Komoyo, G.F.; Yambiyo, B.M.; Manirakiza, A.; Gody, J.C.; Muller, C.P.; Hubschen, J.M.; Nakoune, E.; Snoeck, C.J. Epidemiology and genetic characterization of respiratory syncytial virus in children with acute respiratory infections: Findings from the influenza sentinel surveillance network in Central African Republic, 2015 to 2018. *Health Sci Rep* **2021**, *4*, e298, doi:10.1002/hsr2.298.
244. Asante, I.A.; Hsu, S.N.; Boatemaa, L.; Kwasah, L.; Adusei-Poku, M.; Odoom, J.K.; Awuku-Larbi, Y.; Foulkes, B.H.; Oliver-Commey, J.; Asiedu, E.K.; et al. Repurposing an integrated national influenza platform for genomic surveillance of SARS-CoV-2 in Ghana: a molecular epidemiological analysis. *The Lancet Global Health* **2023**, *11*, e1075-e1085, doi:10.1016/S2214-109X(23)00189-4.
245. Lekana-Douki, S.E.; N'Dilimabaka, N.; Mbongo-Kama, E.; Kandet Yattara, M.; Mintsa Ndong, A.; Ngonga Dikongo, A.M.; Andeko, J.C.; Zong Minko, O.; Koumba Mavoungou, D.S.; Diane, A.; et al. Epidemiology of first cases of SARS-CoV-2 infection, from March to April 2020, in Gabon. *F1000Res* **2022**, *11*, 205, doi:10.12688/f1000research.74378.2.
246. Wolter, N.; Tempia, S.; von Gottberg, A.; Bhiman, J.N.; Walaza, S.; Kleynhans, J.; Moyes, J.; Aitken, S.; Magni, S.; Yun, J.; et al. Healthcare utilization during the first two waves of the COVID-19 epidemic in South Africa: A cross-sectional household survey. *PLoS One* **2023**, *18*, e0290787, doi:10.1371/journal.pone.0290787.
247. Ameme, D.K.; Dadzie, D.; Asiedu-Bekoe, F.; Edu-Quansah, E.P.; Kaburi, B.B.; Wullar, O.; Amo-Mensah, P.; Kenu, E. Influenza A (H1N1)pdm09 outbreak of unknown source in a Ghanaian senior high school. *BMC Public Health* **2020**, *20*, 1423, doi:10.1186/s12889-020-09467-x.

248. Keita, M.B.; Pierre, F.; Ndjomou, J.; Traoré, B.; Tohonamou, P.; Soumaré, M.; Mamadi, S.; Keita, M.A.; Bile, C.E.; Pallawo, R.B.; et al. The first epidemiological and virological influenza surveillance in the Republic of Guinea revealed the predominance of influenza A/H3N2 and B Victoria viruses. *Epidemiology and Infection* **2021**, *149*, e223, doi:10.1017/S0950268821001965.
249. Mahdi, H.A.; Rashid, H.; Qashqari, F.S.; Hariri, S.H.; Marglani, O.A.; Barasheed, O.; Albutti, A.; Alwashmi, A.S.; Shaban, R.Z.; Booy, R.; et al. Syndromic surveillance of respiratory-tract infections and hand hygiene practice among pilgrims attended Hajj in 2021: a cohort study. *BMC Infect Dis* **2022**, *22*, 578, doi:10.1186/s12879-022-07559-0.
250. Mohammed, A.M.; Khalil, M.A.; Fayyadh, R.A. Prevalence and Clinical Features of Ocular Adenoviral Infection among Patients Attended to Ibn-Alhaitham Teaching Eye Hospital in Baghdad, Iraq: A Molecular Study. *Medico-legal update* **2020**, *20*, doi:10.37506/mlu.v20i4.1942.
251. Baaees, M.S.O.; Naiene, J.D.; Al-Waleedi, A.A.; Bin-Azoon, N.S.; Khan, M.F.; Mahmoud, N.; Musani, A. Community-based surveillance in internally displaced people's camps and urban settings during a complex emergency in Yemen in 2020. *Confl Health* **2021**, *15*, 54, doi:10.1186/s13031-021-00394-1.
252. Ministry of Health Seychelles. *Health of our Nation, Annual Health Sector Performance Report, 2018*; 2019.
253. Kumar, N.; Shahul Hameed, S.K.; Babu, G.R.; Venkataswamy, M.M.; Dinesh, P.; Kumar Bg, P.; John, D.A.; Desai, A.; Ravi, V. Descriptive epidemiology of SARS-CoV-2 infection in Karnataka state, South India: Transmission dynamics of symptomatic vs. asymptomatic infections. *EClinicalMedicine* **2021**, *32*, 100717, doi:10.1016/j.eclinm.2020.100717.
254. Elsaid, A.F.; Agrawal, S.; Agrawal, A.; Ghoneum, M. Dietary Supplementation with Biobran/MGN-3 Increases Innate Resistance and Reduces the Incidence of Influenza-like Illnesses in Elderly Subjects: A Randomized, Double-Blind, Placebo-Controlled Pilot Clinical Trial. *Nutrients* **2021**, *13*, doi:10.3390/nu13114133.
255. MacDonald, I.; Hsu, J.L. Epidemiological observations on breaking COVID-19 transmission: from the experience of Taiwan. *J Epidemiol Community Health* **2021**, *75*, 809-812, doi:10.1136/jech-2020-216240.
256. Loevinsohn, G.; Mehoke, T.; Sinywimaanzi P; al, e. Influenza and RSV infection in rural Zambia. **2020**.
